# Shifts in receptors during submergence of an encephalitic arbovirus

**DOI:** 10.1038/s41586-024-07740-2

**Published:** 2024-07-24

**Authors:** Wanyu Li, Jessica A. Plante, ChieYu Lin, Himanish Basu, Jesse S. Plung, Xiaoyi Fan, Joshua M. Boeckers, Jessica Oros, Tierra K. Buck, Praju V. Anekal, Wesley A. Hanson, Haley Varnum, Adrienne Wells, Colin J. Mann, Laurentia V. Tjang, Pan Yang, Rachel A. Reyna, Brooke M. Mitchell, Divya P. Shinde, Jordyn L. Walker, So Yoen Choi, Vesna Brusic, Paula Montero Llopis, Scott C. Weaver, Hisashi Umemori, Isaac M. Chiu, Kenneth S. Plante, Jonathan Abraham

**Affiliations:** 1 Department of Microbiology, Blavatnik Institute, Harvard Medical School, Boston, MA USA; 2 World Reference Center for Emerging Viruses and Arboviruses, University of Texas Medical Branch, Galveston, TX USA; 3 Department of Microbiology and Immunology, University of Texas Medical Branch, Galveston, TX USA; 4 Institute for Human Infections and Immunity, University of Texas Medical Branch, Galveston, TX USA; 5 Department of Immunology, Blavatnik Institute, Harvard Medical School, Boston, MA USA; 6 Department of Neurology, F. M. Kirby Neurobiology Center, Boston Children’s Hospital, Harvard Medical School, Boston, MA USA; 7 MicRoN Core, Harvard Medical School, Boston, MA USA; 8 Department of Medicine, Division of Infectious Diseases, Brigham and Women’s Hospital, Boston, MA USA; 9 Center for Integrated Solutions in Infectious Diseases, Broad Institute of Harvard and MIT, Cambridge, MA USA

**Keywords:** Alphaviruses, Virus-host interactions

## Abstract

Western equine encephalitis virus (WEEV) is an arthropod-borne virus (arbovirus) that frequently caused major outbreaks of encephalitis in humans and horses in the early twentieth century, but the frequency of outbreaks has since decreased markedly, and strains of this alphavirus isolated in the past two decades are less virulent in mammals than strains isolated in the 1930s and 1940s^1–3^. The basis for this phenotypic change in WEEV strains and coincident decrease in epizootic activity (known as viral submergence^3^) is unclear, as is the possibility of re-emergence of highly virulent strains. Here we identify protocadherin 10 (PCDH10) as a cellular receptor for WEEV. We show that multiple highly virulent ancestral WEEV strains isolated in the 1930s and 1940s, in addition to binding human PCDH10, could also bind very low-density lipoprotein receptor (VLDLR) and apolipoprotein E receptor 2 (ApoER2), which are recognized by another encephalitic alphavirus as receptors^4^. However, whereas most of the WEEV strains that we examined bind to PCDH10, a contemporary strain has lost the ability to recognize mammalian PCDH10 while retaining the ability to bind avian receptors, suggesting WEEV adaptation to a main reservoir host during enzootic circulation. PCDH10 supports WEEV E2–E1 glycoprotein-mediated infection of primary mouse cortical neurons, and administration of a soluble form of PCDH10 protects mice from lethal WEEV challenge. Our results have implications for the development of medical countermeasures and for risk assessment for re-emerging WEEV strains.

## Abstract (web summary)

Knockout studies using CRISPR–Cas9 identify PCDH10 as a selective host cellular receptor for western equine encephalitis virus.

## Main

Alphaviruses can cause devastating outbreaks of encephalitis in humans and equids. Examples include WEEV, eastern equine encephalitis virus (EEEV) and Venezuelan equine encephalitis virus^5–7^ (VEEV). The transmission cycle of WEEV involves avian reservoir and amplification hosts and mosquitoes. Humans and horses are affected through the bite of infected mosquitoes. Manifestations of human WEEV infection range from mild or asymptomatic illness to encephalitis, with a case fatality rate as high as 50% in children below one year of age^5,6,8^. Encephalitis can result in permanent neurological sequelae, including seizures, paralysis and intellectual disability^6^. No vaccines or antivirals have been approved by the US Food and Drug Administration for use against WEEV.

WEEV was first isolated during a 1930 outbreak in San Joaquin Valley, California that resulted in around 6,000 cases of encephalitis in horses^7^. The most severe WEEV epidemic occurred in 1941, with more than 3,000 reported human cases^9^. Since the middle of the twentieth century, WEEV outbreaks in humans and equids have markedly declined in frequency and scale^3,9^. Fewer than 700 human cases were documented between 1964 and 2009 in the USA, and seroprevalence in residents of endemic regions in California decreased from 34% in 1960 to less than 3% in 1995^7^. Surveillance of WEEV in birds and mosquitoes also detected a decline in circulation^10^.

Furthermore, the apparent reduction in epizootic infections and enzootic circulation has been accompanied by a decrease in the mammalian virulence of contemporary WEEV strains. Strains isolated in the 1930s and 1940s are more virulent in mouse models and lead to more rapid death than strains isolated later in the century, and a strain isolated in 2005 was shown to be nonpathogenic in mice and Syrian hamsters^2,11^.

In South America, WEEV appeared to decline in a similar manner, with the last major outbreak occurring in 1988 in Argentina^12^, although sporadic spillover events have occurred since^13,14^. However, WEEV re-emerged in Argentina and Uruguay in November 2023, with more than 2,400 equid cases (clinically diagnosed and laboratory confirmed), 103 human cases and 10 human fatalities as of 31 March 2024^12,15–17^. The molecular determinants of WEEV infectivity in mammalian cells remain unknown, as do factors that drive the marked phenotypic change in WEEV strains isolated over the past century. A better understanding of determinants of WEEV infection of mammalian cells is urgently needed for risk assessment of re-emerging strains and to facilitate outbreak preparedness.

## PCDH10 is a WEEV cellular receptor

**Extended Data Fig. 1:**
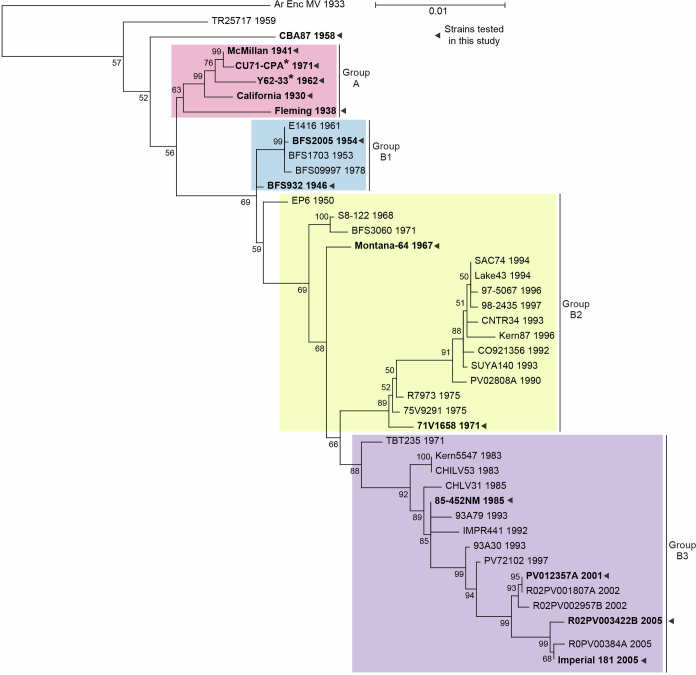
Phylogenetic analysis of WEEV. Maximum likelihood phylogenetic tree of 44 WEEV strains with full genome sequences available, using the coding sequences of the structural polyprotein genes (Supplementary Table 1). The tree is rooted with VEEV strain TC-83 structural polyprotein genes (GenBank MZ399799.1) as an outgroup (not shown). Numbers at nodes indicate bootstrap values. In cases in which the branches are too small bootstrap values may not be shown. Scale bar represents 0.01 nucleotide substitutions per site. Groups A, B1, B2, and B3, are indicated. Taxon labels include strain name and year of isolation. Strains examined in this study are bolded and indicated by a triangle. Asterisks indicate strains reportedly isolated outside of North America.

The alphavirus genome encodes four nonstructural proteins (nsP1–nsP4) and six structural proteins (capsid, E3, E2, 6K, TF and E1). WEEV strains isolated in North America are divided into two lineages: group A and group B^1^ (Extended Data Fig. 1 and Supplementary Table 1). Group B is further subdivided into B1, B2 and B3^1^. Group A contains ancestral WEEV strains, several of which have been demonstrated to be highly pathogenic in mammals^2,9,18^. The B1, B2 and B3 sublineages contain moderately pathogenic strains, and B3 contains at least one strain that is nonpathogenic in animal models^1–3,18^. An evolutionary analysis suggests rapid displacement of the earlier groups by later ones, with B3 being the only group that is known to circulate in North America today^1,3^.

**Fig. 1:**
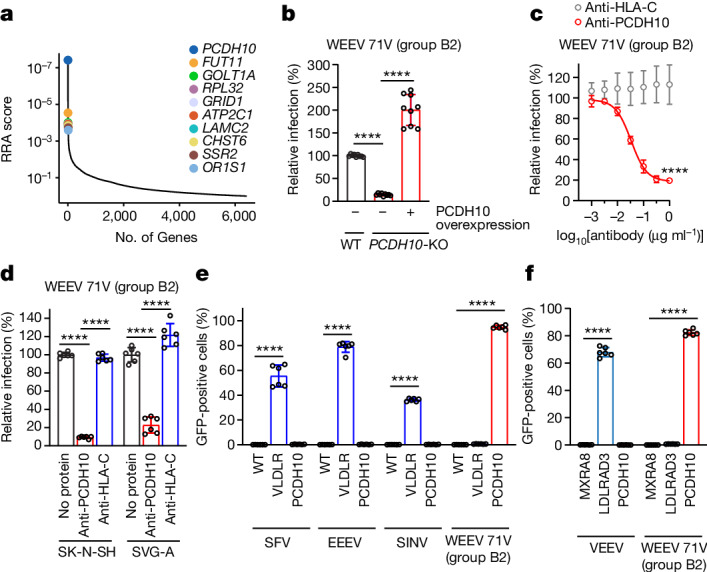
A CRISPR–Cas9 screen identifies PCDH10 as a host factor for WEEV E2–E1-mediated infection. **a**, Results of MAGeCK^20^ analysis for enriched genes in the screen performed using WEEV RVPs (strain 71V) with HEK 293T-Cas9 cells, based on top robust rank aggregation (RRA) scores. **b**, Wild-type (WT) HEK 293T cells, *PCDH10*-knockout (KO) cells or *PCDH10*-knockout cells stably transduced with human PCDH10 (PCDH10 overexpression) were infected with GFP-expressing WEEV RVPs (strain 71V). Infection was measured by flow cytometry. PCDH10 expression was monitored by cell surface immunostaining (Extended Data Fig. 3b). **c**, Infection of HEK 293T cells with WEEV RVPs in the presence of PCDH10 polyclonal antibodies or human leukocyte antigen C (HLA-C) control polyclonal antibodies. Infection was measured by flow cytometry. **d**, Infection of SK-N-SH and SVG-A cells with WEEV RVPs in the presence of PCDH10 polyclonal antibodies or HLA-C control polyclonal antibodies. Infection was measured by flow cytometry. **e**, Wild-type K562 cells or K562 cells stably expressing human VLDLR or human PCDH10 were infected with GFP-expressing RVPs bearing E2–E1 glycoproteins of SFV (SFV4), EEEV (FL91-469), SINV (Toto1101 T6P144) and WEEV (71V), and infection was monitored by flow cytometry. **f**, K562 cells stably expressing human MXRA8, LDLRAD3 or PCDH10 were infected with GFP-expressing RVPs for VEEV (INH-9813) or WEEV (71V). Infection was monitored by flow cytometry. Data are mean ± s.d. from 3 experiments performed in triplicates (*n* = 9) (**b**), 1 experiment performed in triplicates and 1 experiment performed in sextuplets (*n* = 9) (**c**), or 2 experiments performed in triplicates (*n* = 6) (**d**–**f**). One-way ANOVA with Dunnett’s multiple comparisons test (**b**); two-way ANOVA with Šídák’s multiple comparisons test (**c**); two-way ANOVA with Dunnett’s multiple comparisons test (**d**–**f**). ****P < 0.0001. Source Data

**Extended Data Fig. 2:**
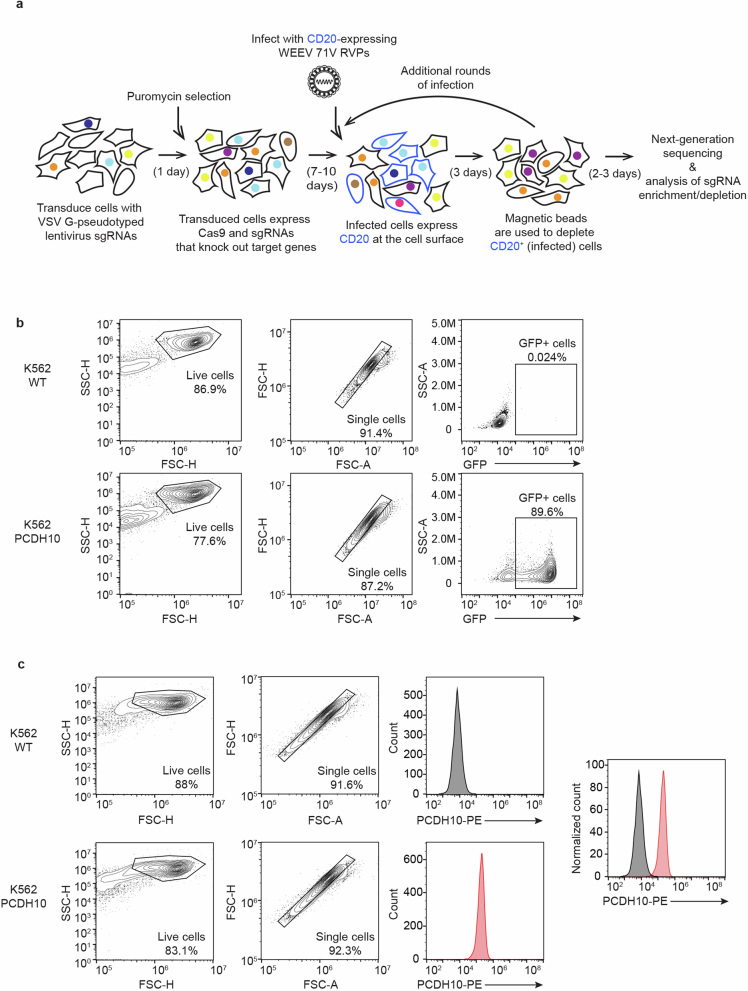
Schematic diagram for CRISPR-Cas9 screen and gating strategy for flow cytometry. **a**, Screening strategy. HEK 293 T cells were transduced with vesicular stomatitis virus glycoprotein (G) pseudotyped lentivirus encoding the sgRNA library. Cells were infected with CD20-expressing WEEV RVPs (strain 71 V), and infected cells were depleted with magnetic beads coated with an anti-CD20 antibody. Uninfected cells were expanded and re-infected iteratively to increase signal-to-noise ratio. Enriched sgRNAs in CD20-negative cells were identified by next-generation sequencing. **b**, Flow cytometry gating strategy for quantifying GFP-expressing cells infected with WEEV RVPs (strain 71 V). Wild-type (WT) K562 cells (upper panels) or K562 cells overexpressing PCDH10 (lower panels) were infected with WEEV RVPs and subjected to flow cytometry analysis. **c**, Flow cytometry gating strategy for monitoring cell surface expression of receptors. Wild-type K562 cells (upper panels) and K562 cells overexpressing human PCDH10 (lower panels) were stained with an anti-PCDH10 antibody, and a PE-conjugated secondary antibody was used for detection. Staining is overlapped in the rightmost panel for comparison.

To identify WEEV cellular receptors on human cells, we performed a pooled CRISPR–Cas9 knockout screen with guide RNAs (10 per gene) targeting membrane-associated proteins in HEK 293T (human kidney epithelial) cells stably expressing Cas9 (Extended Data Fig. 2a). We used a single-cycle reporter virus particle (RVP) system^4^ in which a genomic RNA that encodes the Old World alphavirus Ross River virus nsP1–nsP4, capsid and a reporter were packaged into virions bearing surface glycoproteins of heterologous alphaviruses. For the screen, we chose group B2 WEEV strain 71V1658 (71V), which was originally isolated from an infected horse in Oregon, USA in 1971^19^. This strain does not recognize VLDLR, ApoER2 or low-density lipoprotein receptor (LDLR) class A domain-containing 3 (LDLRAD3), which serve as receptors for other encephalitic alphaviruses^4^. The CRISPR knockout screen identified PCDH10 as the top candidate receptor according to robust rank aggregation^20^ (Fig. 1a and Supplementary Table 2). PCDH10 is a δ2-protocadherin that is expressed in several peripheral tissues, but is notably enriched in the brain, where it participates in synapse development^21–24^. Mutations in *PCDH10* have been linked to autism-spectrum disorders^25^. Protocadherins have no structural similarity to any previously known alphavirus receptors^4,26,27^.

**Extended Data Fig. 3:**
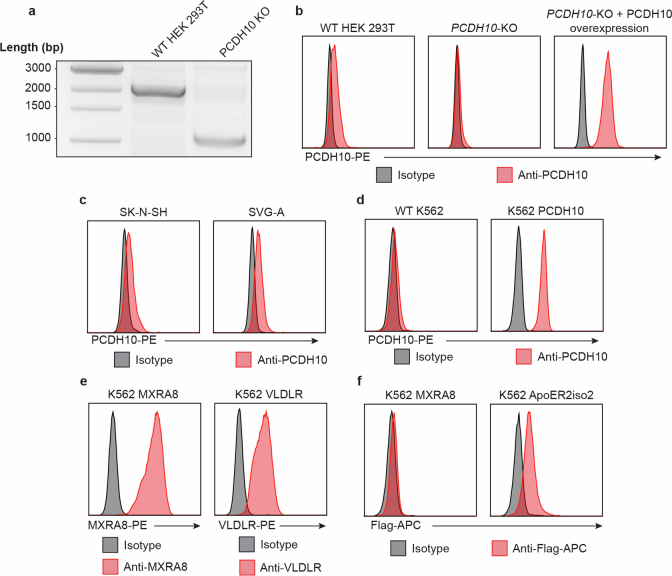
Genotyping and immunostaining to validate cell surface receptor expression. **a**, Genotyping of wild-type (WT) or PCDH10 knockout (KO) HEK 293T clonal cells by PCR. A representative gel from two experiments is shown. **b**, Cell surface staining of PCDH10 for WT HEK 293T cells, clonal PCDH10 KO HEK 293T cells, and clonal PCDH10 KO cells stably transduced to express PCDH10, as monitored by flow cytometry with anti-PCDH10 polyclonal antibodies. PE, *R*-phycoerythrin. **c**, Cell surface staining of SK-N-SH and SVG-A cells with anti-PCDH10 polyclonal antibodies, as monitored by flow cytometry. **d**, Cell surface staining of wild-type K562 cells and K562 cells stably expressing PCDH10, as monitored by flow cytometry. **e**, Cell surface staining of K562 cells stably expressing human MXRA8 or VLDLR, as monitored by flow cytometry. **f**, Cell surface staining of K562 cells stably expressing human MXRA8 or ApoER2 (iso2) using RAP-Flag as a substitute for primary antibody and APC-conjugated anti-Flag antibody as a secondary antibody. Staining was monitored by flow cytometry. Gel source data for panel **a** are provided in Supplementary Fig. 1.

Clonal PCDH10-knockout HEK 293T cells became resistant to infection by WEEV 71V RVPs encoding GFP, and infection could be rescued by PCDH10 overexpression (Fig. 1b and Extended Data Fig. 3a,b). Infection by WEEV 71V RVPs of HEK 293T cells, SK-N-SH (a human neuroblast cell line) and SVG-A (a human astrocyte cell line) could also be blocked by polyclonal antibodies against PCDH10, but not by an isotype control antibody (Fig. 1c,d and Extended Data Fig. 3c). K562 cells, a human lymphoblast cell line^28^ that is refractory to entry of all tested alphaviruses^4^, do not express PCDH10 on their surface (Extended Data Fig. 3d). PCDH10 overexpression resulted in robust WEEV 71V RVP infection of these cells (Fig. 1e). Overexpression of VLDLR, a receptor for EEEV, Semliki Forest virus (SFV) and Sindbis virus^4^ (SINV) or of LDLRAD3, a VEEV receptor^26^, did not affect WEEV 71V RVP entry (Fig. 1e,f and Extended Data Fig. 3e).

## WEEV interacts with PCDH10 EC1

**Fig. 2:**
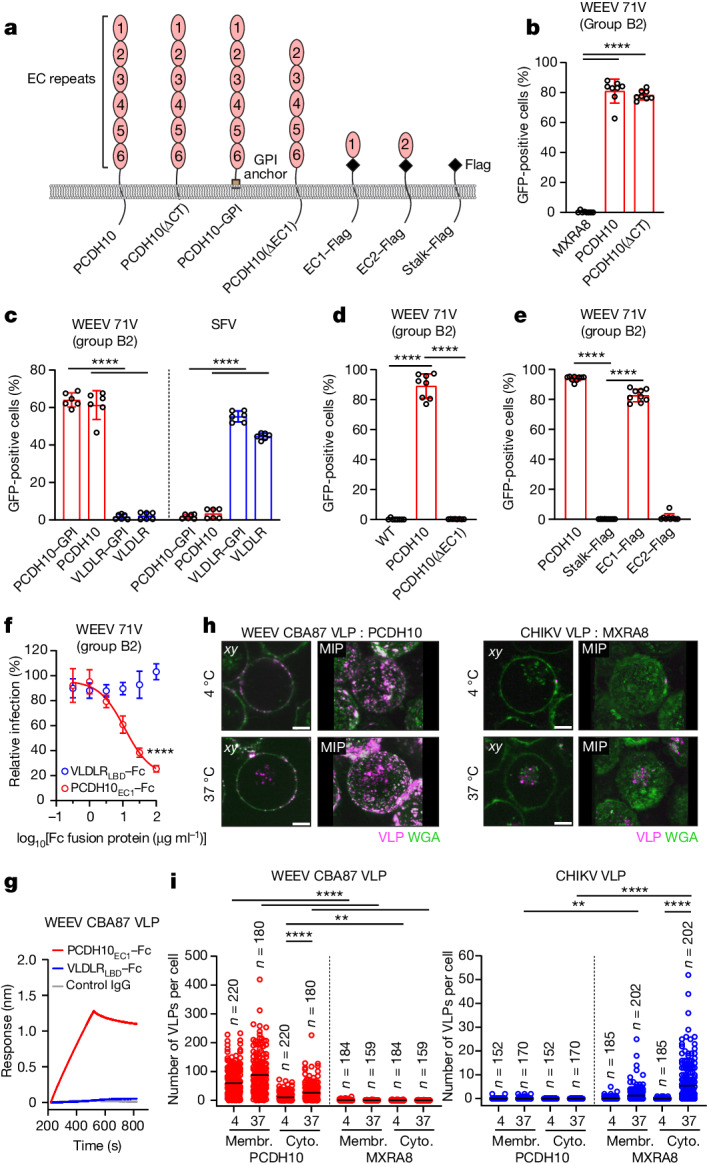
PCDH10 binds WEEV E2–E1 through extracellular cadherin repeat 1 and mediates cellular attachment and internalization. **a**, Schematic diagram of PCDH10 constructs: cytoplasmic tail deletion construct (PCDH10(ΔCT)), GPI-anchored construct (PCDH10–GPI), EC1 deletion construct (PCDH10(ΔEC1)), single EC1 or EC2 constructs with internal Flag tags and Flag-tagged stalk-only construct (stalk–Flag). **b**–**e**, K562 cells expressing PCDH10 or PCDH10(ΔCT) (**b**), PCDH10–GPI, VLDLR or VLDLR–GPI (**c**), PCDH10(ΔEC1) (**d**) or stalk–Flag, EC1–Flag or EC2–Flag (**e**) were infected with the indicated GFP-expressing RVPs. Infection was monitored by flow cytometry. **f**, HEK 293T cells were infected with WEEV 71V RVPs pre-incubated with VLDLR_LBD_–Fc or PCDH10_EC1_–Fc at the indicated concentrations. Infection was measured by flow cytometry. **g**, Biolayer interferometry sensorgram of WEEV CBA87 VLP binding to tips coated with VLDLR_LBD_–Fc, PCDH10_EC1_–Fc, or a control IgG. One representative sensorgram from two experiments is shown. **h**, *xy* slice and maximum intensity projection (MIP) of representative images of wheat germ agglutinin (WGA)-stained K562 cells ectopically expressing human PCDH10 or MXRA8 co-incubated with fluorescently labelled WEEV CBA87 VLPs (left) or CHIKV 37997 strain VLPs (right) imaged at the indicated temperatures (see Extended Data Fig. 5). Scale bars, 5 μm. **i**, Number of WEEV CBA87 or CHIKV 37997 VLPs bound to cell membranes (membr.) or internalized into the cytoplasm (cyto.) of individual K562 cells expressing human PCDH10 or MXRA8 at the indicated temperatures (see Extended Data Fig. 6). Data are mean from 2 experiments with numbers of individual cells indicated (**i**), mean ± s.d. from 2 experiments performed in triplicates (*n* = 6) (**c**,**f**), 3 experiments performed in duplicates or triplicates (*n* = 8) (**b**,**d**), or 3 experiments performed in triplicates (*n* = 9) (**e**). Two-way ANOVA with Šídák’s multiple comparisons test: **P = 0.0054 (WEEV CBA87 VLP), **P = 0.0039 (CHIKV VLP) (**i**); two-way ANOVA with Šídák’s multiple comparisons test (**f**); two-way ANOVA with Dunnett’s multiple comparisons test (**c**). one-way ANOVA with Dunnett’s multiple comparisons test (**b**–**e**). Source Data

**Extended Data Fig. 4:**
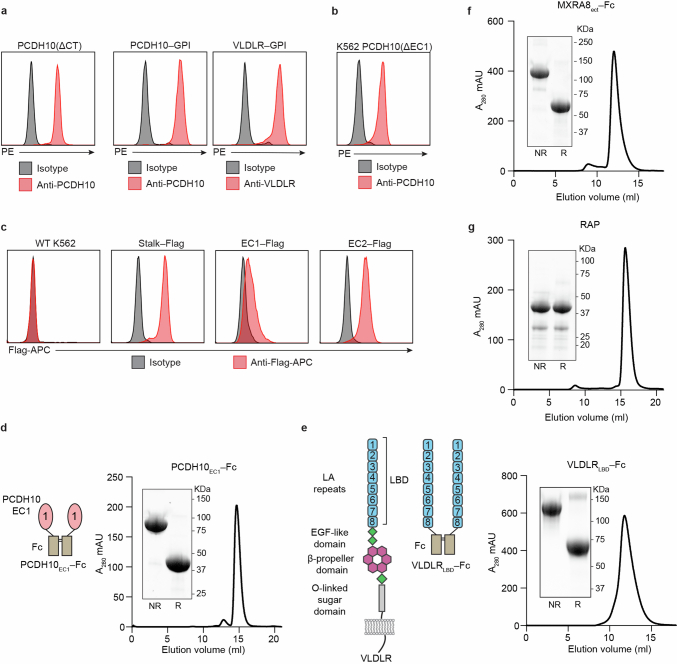
Cell surface staining for PCDH10 constructs and size exclusion chromatography for recombinant proteins. **a**, Cell surface staining of K562 cells stably expressing human PCDH10(ΔCT), PCDH10-GPI, or VLDLR-GPI, as monitored by flow cytometry. **b**, Cell surface staining of K562 cells stably expressing PCDH10(ΔEC1), as monitored by flow cytometry. **c**, Cell surface staining of K562 cells stably expressing stalk-Flag, EC1-Flag, or EC2-Flag, as monitored by flow cytometry. **d**–**e**, Schematic diagrams and size exclusion chromatography traces of Fc fusion proteins, PCDH10_EC1_-Fc (**d**) and VLDLR_LBD_-Fc (**e**). A schematic diagram of full-length VLDLR is also included in **e**. Insets are SDS-PAGE gels of pooled peak fractions, visualized using a stain-free system. A representative gel from at least two experiments is shown. **f**–**g**, Size exclusion chromatography traces of MXRA8_ect_-Fc (**f**) and RAP (**g**); insets are SDS-PAGE gels of pooled peak fractions, visualized using a stain-free system. A representative gel from at least two experiments is shown. Size exclusion chromatography experiments were performed at least three times and representative gels and traces are shown. NR, nonreducing; R, reducing. Gels source data for panels **d**–**g** are provided in Supplementary Fig. 1.

PCDH10 contains six extracellular cadherin repeats (EC1–EC6) connected by loops rigidified by calcium ion coordination. A stalk connects the ectodomain to a transmembrane helix, followed by a long cytoplasmic tail (Fig. 2a). The cytoplasmic tails and transmembrane helices of two other alphavirus receptors, LDLRAD3 and matrix remodelling-associated protein 8 (MXRA8), are dispensable for alphavirus entry^26,27^. We generated a PCDH10 construct, removing the cytoplasmic tail (PCDH10(ΔCT)) or replacing the cytoplasmic tail and the transmembrane helix with a glycophosphatidylinositol (GPI) anchor (Fig. 2a and Extended Data Fig. 4a). We also generated a GPI-anchored VLDLR construct (VLDLR–GPI) and RVPs for SFV, an alphavirus that uses VLDLR as a receptor^4^. PCDH10(ΔCT) supported WEEV 71V RVP entry (Fig. 2b). PCDH10–GPI, but not VLDLR–GPI, supported WEEV 71V RVP entry, and the opposite was true of SFV RVPs (Fig. 2c). Thus, the PCDH10 cytoplasmic tail and transmembrane segments are not required for WEEV cellular entry.

Owing to the expected rigidity of the PCDH10 ectodomain, we hypothesized that the most membrane-distal repeats (EC1 or EC2) would be the most likely sites of WEEV attachment. A PCDH10 construct in which EC1 is deleted could not support WEEV 71V RVP infection when ectopically expressed on K562 cells, suggesting that WEEV binds PCDH10 EC1 (Fig. 2a,d and Extended Data Fig. 4b). We generated single extracellular cadherin constructs in which the ectodomain was replaced by either EC1 or EC2 (Fig. 2a). Overexpression of the single EC1, but not the single EC2 construct or a stalk-only control construct, rendered K562 cells susceptible to infection by WEEV 71V RVPs (Fig. 2e and Extended Data Fig. 4c).

We tested whether an Fc fusion protein containing EC1 (PCDH10_EC1_–Fc) (Extended Data Fig. 4d) could block WEEV 71V RVP entry into cells. As a control, we included an Fc fusion protein containing the VLDLR ligand-binding domain (LBD) (VLDLR_LBD_–Fc) (Extended Data Fig. 4e), which contains binding sites for EEEV, SFV and SINV^4^. We found that PCDH10_EC1_–Fc but not VLDLR_LBD_–Fc blocked WEEV 71V RVP infection of HEK 293T cells (Fig. 2f).

Using biolayer interferometry, we tested whether PCDH10_EC1_–Fc could directly bind purified WEEV virus-like particles (VLPs). The source sequence strain for the WEEV VLPs (CBA87^29^) was isolated in 1958 from an infected horse in Córdoba, Argentina^11^. This strain has been used in investigational VLP-based vaccines against encephalitic alphaviruses modified for high-yield expression^29^. Sensor tips coated with PCDH10_EC1_–Fc, but not VLDLR_LBD_–Fc or a control human IgG, bound WEEV CBA87 VLPs (Fig. 2g). Thus, PCDH10 EC1 is the site of WEEV attachment.

## PCDH10 mediates attachment and uptake

**Extended Data Fig. 5:**
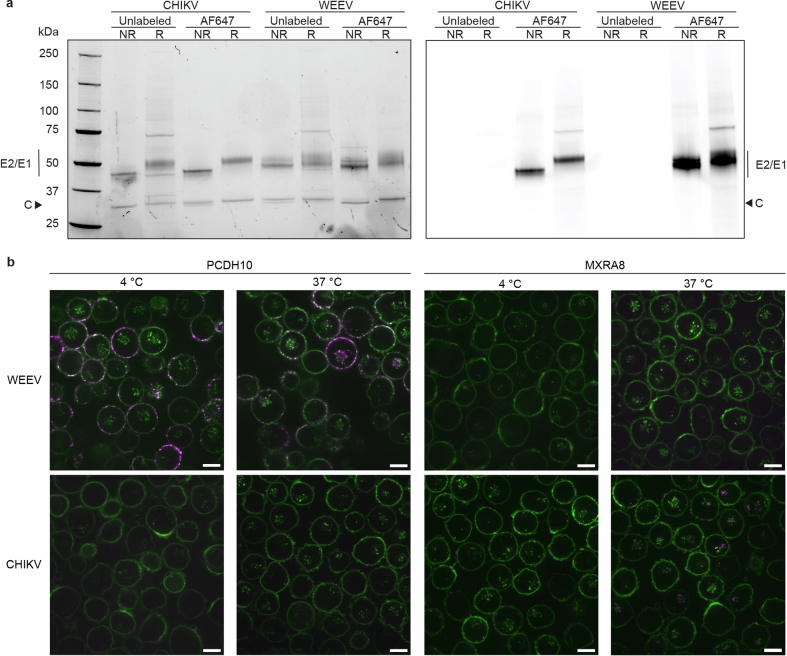
Gels of purified VLPs and representative confocal images for VLP cell attachment and internalization. **a**, SDS-PAGE gels of unlabeled or AF647-labeled CHIKV and WEEV VLPs, visualized using a stain-free system (left) or lasers to excite the fluorophore (right). As capsid proteins are protected within the viral membrane, lack of AF647 conjugation to the capsid proteins indicates that VLP membranes are intact. The experiment was performed three times and representative gels are shown. Positions of the E2–E1 glycoproteins and capsid (C) are indicated. NR, nonreducing; R, reducing. **b**, K562 cells stably expressing human PCDH10 or human MXRA8 were incubated with fluorescently labeled VLPs at 4 °C or 37 °C and then imaged by live cell confocal microscopy. WGA: wheat germ agglutinin. Scale bar is 10 μm. The experiment was performed twice, and representative images are shown. Gel source data for panels **a** and **b** are provided in Supplementary Fig. 1.

**Extended Data Fig. 6:**
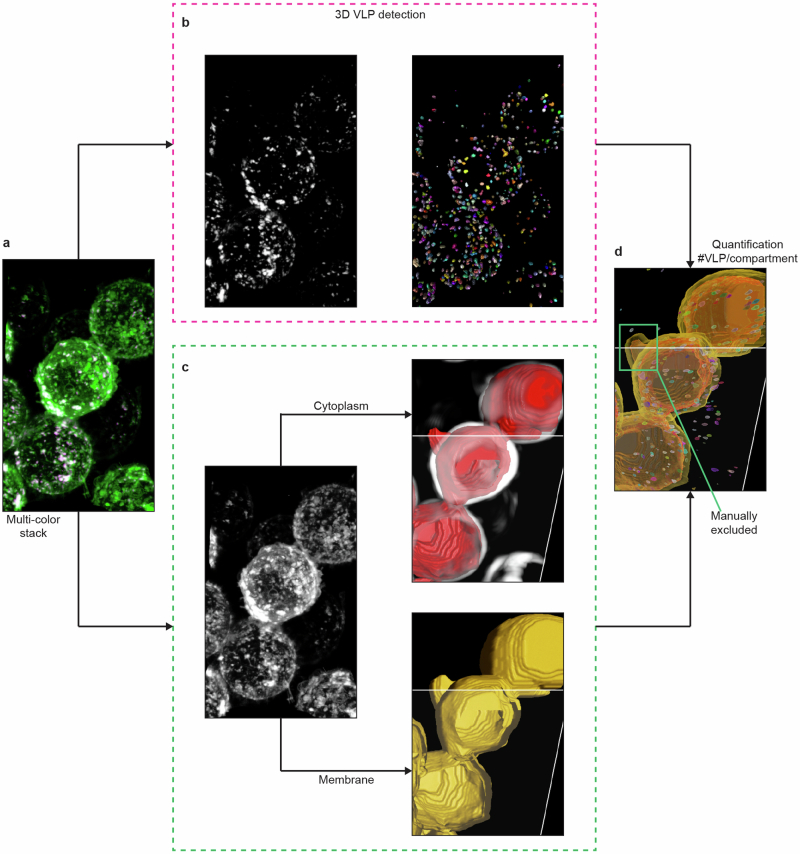
Workflow diagram of 3D quantification of VLP cell attachment and internalization. **a**, 3D analysis of multi-color stacks (magenta, VLPs; green, cell membranes) using Arivis 4DFusion. Two custom-made pipelines were used to detect VLPs and cellular compartments. **b**, 3D rendering of VLP stacks (left) and 3D rendering of detected VLPs (right). **c**, 3D rendering of cellular membranes stacks (left), 3D rendering of the detected cytoplasms (red) overlayed with an enhanced-membrane filter (white) (right, top), and 3D rendering of the detected membranes (yellow) (right, bottom). Objects obtained in each pipeline were combined to quantify the number of VLPs in each cellular compartment. **d**, 3D rendering of detected objects from the two pipelines combined, used to quantify the number of VLPs in each cellular compartment. Related to Fig. 2i. An example of small objects that could result from non-intact cells at the edge of the imaged volume, or cellular blebs segmented as complete cells, is indicated in the green box. These objects were removed by a volume filter and the VLPs they contain were not counted. See Methods for additional details.

We tested whether PCDH10 could support cell surface attachment and internalization of WEEV VLPs (strain CBA87) using live-cell confocal microscopy. As a control, we included VLPs for chikungunya virus (CHIKV), an Old World alphavirus that recognizes human MXRA8 as a receptor^27^. We incubated fluorescently labelled VLPs with transduced K562 cells and found that expression of human PCDH10, but not human MXRA8, allowed WEEV VLP binding to cell surface membranes (Fig. 2h,i and Extended Data Figs. 5 and 6). An increase in particles in the cytoplasm of cells was detected at 37 °C versus 4 °C, suggesting internalization. Conversely, ectopic expression of human MXRA8, but not human PCDH10, increased the number of CHIKV VLPs in the cytoplasm of cells at 37 °C versus 4 °C. Thus, PCDH10 can bind WEEV E2–E1 to mediate virus cell surface attachment and internalization.

## VLDLR and ApoER2 as alternative receptors

Our phylogenetic analysis using the structural polyprotein coding sequences of 44 WEEV strains collected over 90 years matched a previous study by Bergren et al.^1^ that used the full-length genomic sequences of 33 strains (Extended Data Fig. 1 and Supplementary Table 1). We included the group A ancestral strain California, a WEEV strain retrieved from a horse (San Joaquin Valley, 1930), and the Fleming and McMillan strains, isolated from human cases (1938 and 1941, respectively). We also included two strains that were not examined by Bergren et al.: Y62-33 and CU71-CPA, which are closely related to group A strains and are thought to originate outside of North America, bringing their origins into question. The South American isolates of WEEV that we examined are phylogenetically distinct from North American lineages (Extended Data Fig. 1).

The E2 subunit of the E2–E1 spike protein typically contains binding sites for cellular receptors^30–33^. Among viral proteins, WEEV E2 sequences vary most in strains that differ in virulence in mammals^1^, and E2 substitutions have been implicated in causing pathogenicity differences between McMillan, a virulent group A strain, and Imperial 181, a group B3 strain that appears to have lost pathogenicity in mammals^18^. To test whether WEEV recognition of PCDH10 may have drifted during viral evolution, we tested whether RVPs bearing E2–E1 spike proteins of select WEEV strains representing groups A, B1, B2 and B3 (14 in total) differ in receptor dependencies using K562 cells expressing human PCDH10. We also included K562 cells that express human VLDLR and ApoER2 because they serve as cellular receptors for EEEV^4^, another encephalitic alphavirus, and used the shorter isoform of ApoER2, which does not contain LDLR class A repeats 4–7 and is a presumed dominant form^34,35^ (Extended Data Fig. 3f).

**Fig. 3:**
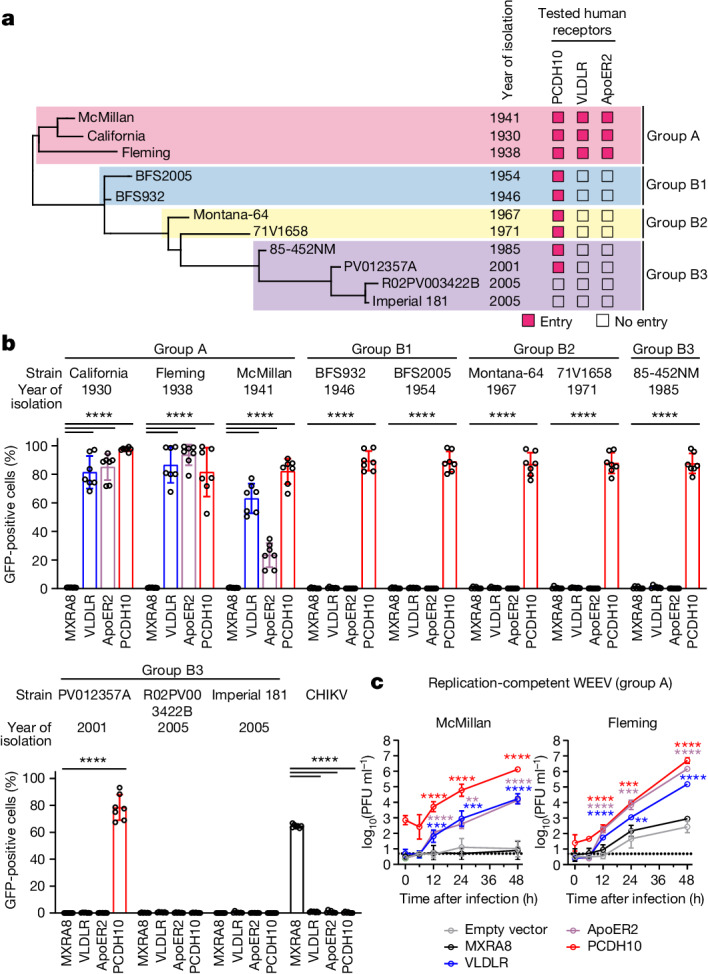
Shifts in human receptor recognition during WEEV spike protein evolution. **a**, Partial phylogenetic tree of WEEV strains and summary of infectivity assays with GFP-expressing RVPs for various strains of WEEV in K562 cells stably overexpressing the indicated proteins. Infectivity assays and full phylogenetic tree are in **b** and Extended Data Fig. 1. **b**, K562 cells stably expressing human orthologues of MXRA8, VLDLR, ApoER2 or PCDH10 were infected with GFP-expressing RVPs for the indicated strains of WEEV. Infection was quantified by flow cytometry. See Extended Data Fig. 7a for infectivity assay on additional strains. **c**, Viral replication for WEEV McMillan and Fleming in transduced K562 cells, multiplicity of infection (MOI) = 0.01. Data are mean ± s.d. from 3 experiments performed in duplicates or triplicates (*n* = 7) (**b**), or two experiments performed in triplicates (*n* = 6) (**c**). Two-way ANOVA with Dunnett’s multiple comparisons test compared with MXRA8 (**b**) or empty vector (**c**). **c**, McMillan 12 h VLDLR, ***P = 0.0008; 24 h ApoER2, **P = 0.0019; 24 h VLDLR, ***P = 0.0005; Fleming 24 h VLDLR, **P = 0.0055; 24 h ApoER2, ***P = 0.0002; 24 h PCDH10, ***P = 0.0005. Source Data

**Extended Data Fig. 7:**
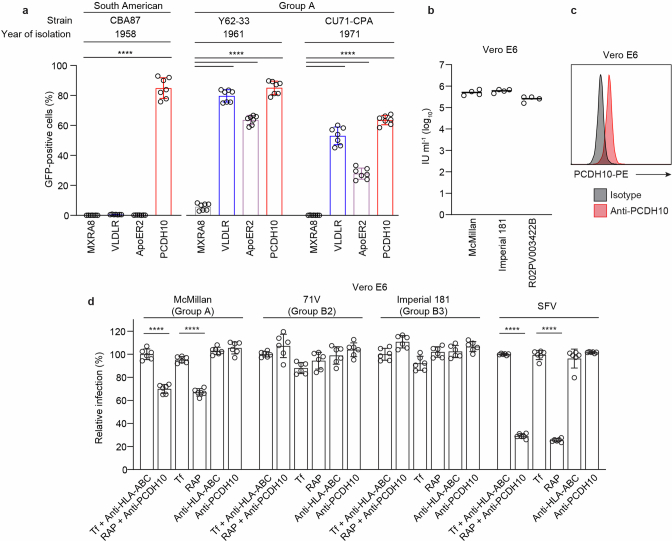
Variation in human receptor recognition by different strains of WEEV. **a**, K562 cells expressing human MXRA8, VLDLR, ApoER2, or PCDH10 were infected with GFP-expressing RVPs of WEEV CBA87, Y62-33 or CU71-CPA strains. Infection was monitored by flow cytometry. **b**, Titration of GFP-expressing RVPs on Vero E6 cells. Titers are represented as infectious units per milliliter (IU ml^−1^) determined by limiting dilution method in infected cells 24 h post infection. See Methods for additional details. **c**, Cell surface staining for PCDH10 on Vero E6 cells as monitored by flow cytometry. **d**, Vero E6 cells were infected with GFP-expressing RVPs of WEEV McMillan, 71 V, or Imperial 181, in the presence or absence of anti-PCDH10 antibodies, RAP, or both. SFV was used as a control RVP. Anti-HLA-ABC antibodies and transferrin (Tf) were used as control proteins. Infection was monitored by flow cytometry. Data are mean from two experiments performed in duplicates (n = 4) (**b**), or mean ± s.d. from two experiments performed in triplicates (n = 6) (**d**), or three experiments performed in duplicates or triplicates (n = 7) (**a**). Two-way ANOVA with Dunnett’s multiple comparisons test, ****P < 0.0001 (**a**, **d**). Source Data

Remarkably, group A strains California (1930), Fleming (1938) and McMillan (1941) could engage not only PCDH10, but also VLDLR and ApoER2 to infect K562 cells (Fig. 3a,b). We observed the same receptor dependencies for the group A strains Y62-33 and CU71-CPA (Extended Data Fig. 7a). All RVPs tested for group B1 and B2 strains and South American strain CBA87 recognized PCDH10, but not VLDLR or ApoER2 (Fig. 3a,b and Extended Data Fig. 7a). Group B3 strains isolated in 1985 and 2001 recognized PCDH10, but two group B3 strains from 2005 did not: Imperial 181, isolated from mosquitoes in California, and R02PV003422B, isolated from mosquitoes in Texas (Fig. 3a,b and Supplementary Table 1). To confirm that RVPs for these two strains had been generated successfully, we used them to infect Vero E6 cells. They yielded similar levels of infection as McMillan strain (group A) RVPs (Extended Data Fig. 7b).

**Extended Data Fig. 8:**
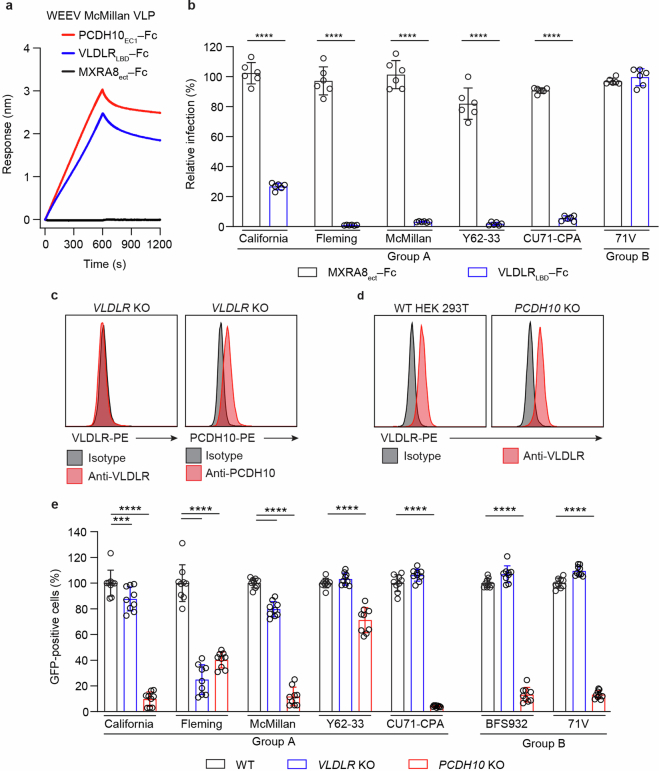
Functional assessment of Group A WEEV strain interactions with PCDH10 and VLDLR. **a**, Biolayer interferometry sensorgram of WEEV McMillan VLP binding to sensor tips coated with human VLDLR_LBD_-Fc, PCDH10_EC1_-Fc, or MXRA8_ect_-Fc. The experiment was performed twice, and a representative sensorgram is shown. **b**, K562 cells stably expressing human PCDH10 were infected with GFP-expressing RVPs in the presence of 31.6 µg ml^−1^ of VLDLR_LBD_-Fc or MXRA8_ect_-Fc. Infection was measured by flow cytometry. **c**, Cell surface staining of VLDLR and PCDH10 for clonal VLDLR KO HEK 293T cells, as monitored by flow cytometry. **d**, Cell surface staining of VLDLR for wild-type or clonal PCDH10 KO HEK 293T cells, as monitored by flow cytometry. **e**, Wild-type, VLDLR KO, or PCDH10 KO HEK 293T cells were infected with GFP-expressing RVPs of the indicated WEEV strains. Infection was measured by flow cytometry. Data are mean ± s.d. from two experiments performed in triplicates (n = 6) (**b**), or three experiments performed in triplicates (n = 9) (**e**). Two-way ANOVA with Šídák’s multiple comparisons test, ****P < 0.0001 (**b**). Two-way ANOVA with Dunnett’s multiple comparisons test, ***P = 0.0005, ****P < 0.0001 (**e**). Source Data

To confirm that PCDH10, VLDLR and ApoER2 can support infection by group A WEEV strains, we rescued replication-competent WEEV Fleming and McMillan from molecular clones, and found they replicated faster and to higher levels in K562 cells expressing PCDH10, VLDLR or ApoER2 than in control cells (Fig. 3c). Alphaviruses usually interact with LDLR-related proteins through the receptors’ LBD^4,31–33^. To test whether group A strain WEEV spike proteins interact with the LBD of VLDLR, we purified McMillan VLPs. In biolayer interferometry binding experiments, unlike results with WEEV CBA87 VLPs, sensor tips coated with VLDLR_LBD_–Fc and PCDH10_EC1_–Fc bound WEEV McMillan VLPs (Extended Data Fig. 8a).

In entry-blocking assays with soluble receptor–Fc fusion proteins using K562 cells ectopically expressing PCDH10, VLDLR_LBD_–Fc blocked infection by all five group A strains (Extended Data Fig. 8b), suggesting that these strains bind VLDLR in a manner that competes with PCDH10 binding. In the same assays, infection by group B2 strain 71V, which only uses PCDH10 as a receptor, was unaffected, and an Fc fusion protein comprising the ectodomain of human MXRA8 (MXRA8_ect_–Fc), used as a negative control, had no effect on viral entry (Extended Data Figs. 4f and 8b).

Because WEEV strains may differ in the efficiency with which they use each alternative cellular receptor, we tested the effect of genetic disruption of PCDH10 or VLDLR on RVP entry for group A strains on HEK 293T cells. HEK 293T cells express PCDH10 and VLDLR (Extended Data Figs. 3b and  8c,d) but do not express ApoER2^4^. RVPs for California, McMillan and CU71-CPA were impacted in clonal PCDH10-knockout HEK 293T cells but not in clonal VLDLR-knockout HEK 293T cells, suggesting that they depend on PCDH10 to infect HEK 293T cells (Extended Data Fig. 8e). Fleming was only partially affected by PCDH10 knockout or VLDLR knockout, whereas Y62-33 was minimally affected by knockout of either receptor (Extended Data Fig. 8e). Thus, Fleming and Y62-33 are likely to use PCDH10 and VLDLR with similar efficiency on this cell type. Group B strains BFS932 and 71V, which can bind PCDH10 but not VLDLR, were affected by PCDH10 knockout but not by VLDLR knockout.

## Receptors determine WEEV neurotropism

**Extended Data Fig. 9:**
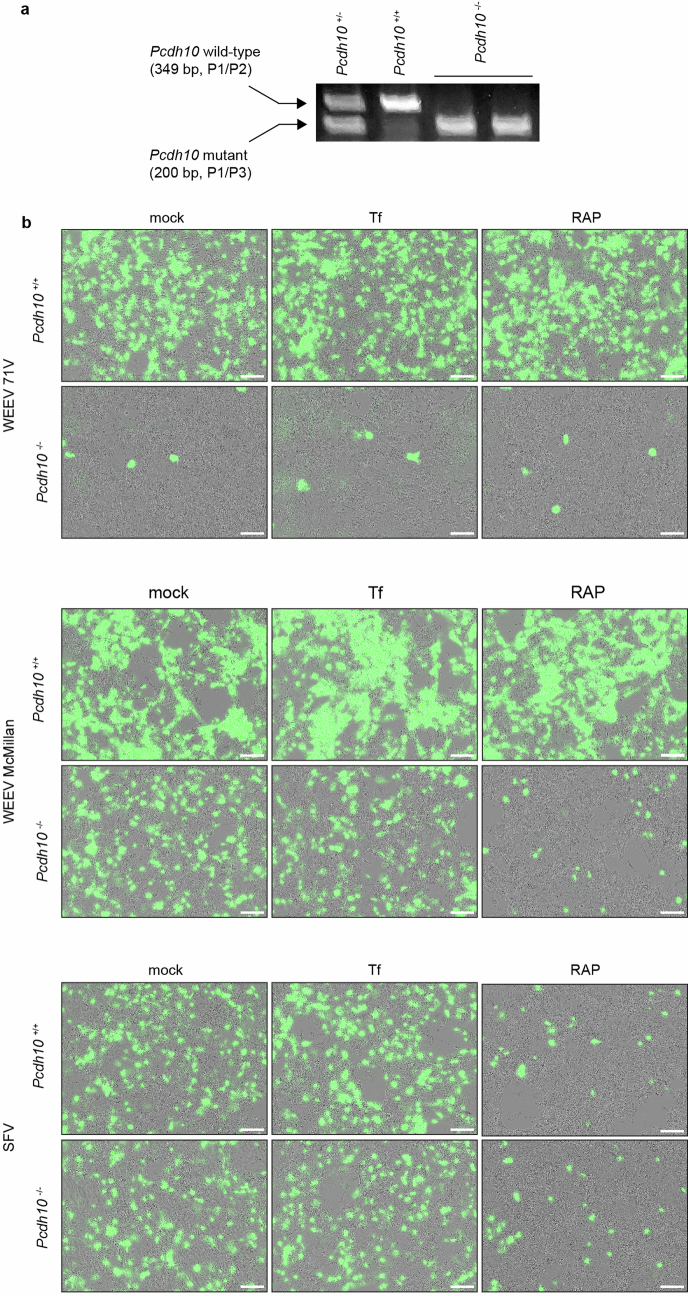
Mouse genotyping and images for infectivity studies in primary murine cortical neurons. **a**, Genotyping PCR for two PCDH10 KO (*Pcdh10*^−/−^) mice used in Fig. 4a,b. Examples of genotyping PCR for wild-type (*Pcdh10*^+/+^) and heterozygote (*Pcdh10*^+/−^) littermates are shown for reference. P1, P2, and P3 are PCR primers used for the reaction (see Methods for additional information). Band patterns for genotyping PCR of *Pcdh10*^−/−^ mice are interpreted as previously established^21^. Genotyping was performed once for each pup. **b**, Merged phase contrast and fluorescent channels for images shown in Fig. 4a, as well as representative images for the mock treatment groups. Images are representative of two independent experiments. Scale bars are 100 µm. Gel source data for panel **a** are provided in Supplemental Fig. 1.

PCDH10, VLDLR and ApoER2 are expressed on cells in the central nervous system^21,22,36^. We tested whether PCDH10 and receptors in the LDLR family could redundantly direct neurotropism of group A WEEV strains and whether PCDH10 is the determinant of neurotropism for group B strains that recognize only this receptor. We infected cortical neurons isolated from postnatal day 1 or day 2 wild-type and *Pcdh10*^−/−^ mice^21^ (Extended Data Fig. 9a) with WEEV RVPs for the group A strain McMillan and the group B strain 71V. We included RVPs for SFV, which depend only on LDLR-family receptors to infect mouse cortical neurons^4^, as a control virus. Infection was performed in the absence or presence of receptor-associated protein (RAP) (Extended Data Fig. 4g), a near-universal ligand antagonist for LDLR family members that can block alphavirus E2–E1 spike protein binding to VLDLR or ApoER2^4,37,38^.

**Fig. 4:**
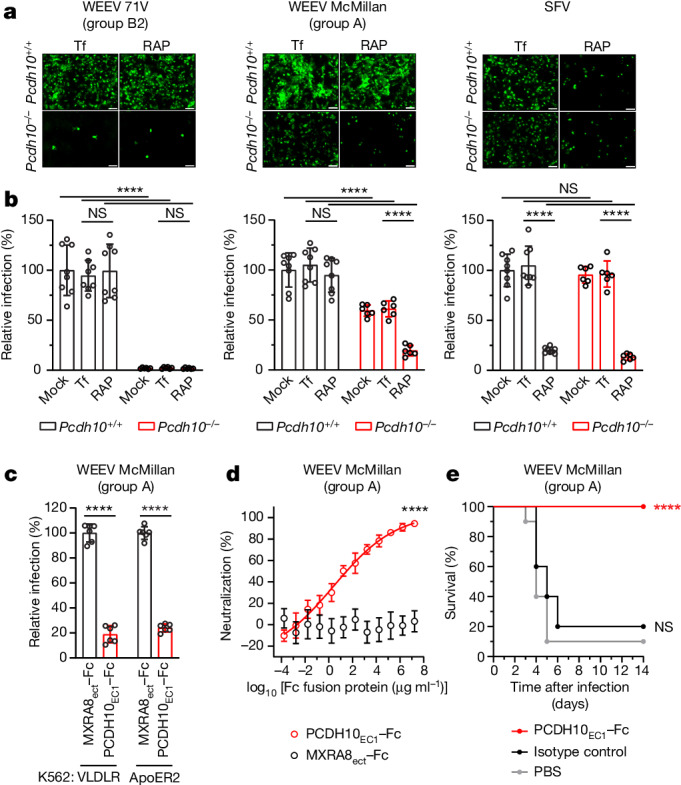
PCDH10 is a determinant of WEEV neurotropism and pathogenicity. **a**, Cortical neurons isolated from wild-type (*Pcdh10*^+/+^) or PCDH10-knockout (*Pcdh10*^−/−^) C57BL/6J mice on postnatal day 1 or 2 were infected with GFP-expressing RVPs bearing E2–E1 spike proteins of WEEV 71V, WEEV McMillan or SFV in the presence of 100 µg ml^−1^ RAP or transferrin (Tf; control). Representative images taken at 24 h post-infection. Scale bars, 100 µm. **b**, Quantification of infection of wild-type or *Pcdh10*^−/−^ mouse cortical neurons in **a** using a live-cell imaging system. Relative infection is normalized to infection levels in wild-type neurons without transferrin or RAP. See Methods for additional details. **c**, K562 cells expressing VLDLR or ApoER2 were infected with GFP-expressing WEEV McMillan RVPs in the presence of 316 µg ml^−1^ MXRA8_ect_–Fc or PCDH10_EC1_–Fc. Infection was quantified by flow cytometry. **d**, WEEV McMillan plaque reduction neutralization assay with the indicated proteins performed on Vero E6 cells. **e**, Six-week-old CD1 mice were administered PCDH10_EC1_–Fc fusion protein, an isotype control antibody, or phosphate-buffered saline (PBS) intraperitoneally 6 h before subcutaneous inoculation with 1,000 PFU of WEEV McMillan rescued from a molecular clone. Survival of the mice was monitored daily. Infection of mouse cortical neurons was performed in two independent experiments, each consisting of neurons from two wild-type mice and one *Pcdh10*^−/−^ mouse. Data are mean ± s.d. **b**, *Pcdh10*^+/+^, *n* = 8; *Pcdh10*^−/−^ *n* = 6; Two-way ANOVA with Tukey’s multiple comparisons test. **c**,**d**, Data are mean ± s.d. from 2 experiments performed in triplicates (*n* = 6). Two-way ANOVA with Dunnett’s multiple comparisons test (**c**) or Šídák’s multiple comparisons test (**d**). **e**, For PCDH10_EC1_–Fc protection experiment: PBS, *n* = 10; PCDH10_EC1_–Fc, *n* = 10; isotype control, *n* = 10 mice. Log-rank (Mantel–Cox) test comparing PCDH10_EC1_–Fc or isotype control to PBS. NS, not significant. Source Data

WEEV 71V RVPs robustly infected wild-type neurons, but infection was almost completely abolished in *Pcdh10*^−/−^ neurons (Fig. 4a,b and Extended Data Fig. 9b). RAP addition did not affect WEEV 71V RVP infection of wild-type neurons. Thus, for a WEEV strain that only recognizes PCDH10 and not LDLR-family receptors, PCDH10 is the sole determinant of neuronal infection. McMillan RVPs robustly infected wild-type neurons; however, infection was reduced but not abolished in *Pcdh10*^−/−^ neurons (Fig. 4a,b and Extended Data Fig. 9b), suggesting that LDLR-related proteins could compensate for the absence of PCDH10 on neurons. Addition of RAP further reduced McMillan RVP infection of *Pcdh10*^−/−^ neurons (Fig. 4a,b and Extended Data Fig. 9b), demonstrating that LDLR-family receptors are likely to mediate McMillan entry into neurons in the absence of PCDH10. Conversely, RAP treatment did not inhibit McMillan RVP infection of wild-type neurons (Fig. 4a,b and Extended Data Fig. 9b), indicating that access to PCDH10 as a receptor was sufficient for infection. As expected, RAP blocked SFV infection of both genotypes of primary cortical neurons (Fig. 4a,b and Extended Data Fig. 9b).

Collectively, these results show that PCDH10 and LDLR-family receptors redundantly support neurotropism of the McMillan strain and that 71V, a group B2 strain that does not bind LDLR-family members, depends on PCDH10 as a primary receptor on mouse cortical neurons.

## Decoy protects against lethal challenge

Our finding that VLDLR_LBD_–Fc could block WEEV McMillan RVP infection of K562 cells stably expressing PCDH10 suggests that group A strains recognize PCDH10 and VLDLR through an overlapping surface on their E2–E1 spike proteins (Extended Data Fig. 8b). Additionally, we found that PCDH10_EC1_–Fc could block McMillan RVP infection of K562 cells expressing VLDLR or ApoER2 (Fig. 4c) and infection of Vero E6 cells by replication-competent WEEV McMillan (Fig. 4d), confirming that PCDH10_EC1_–Fc can block access to multiple receptors that redundantly support WEEV infection.

We chose the WEEV McMillan strain for in vivo experiments because it is the most well-studied group A strain in vivo^2,11,18^. Subcutaneous challenge with McMillan strain is lethal in CD1 mice, with most if not all animals succumbing within five days^2^. When five- to six-week-old CD1 mice were subcutaneously inoculated in the footpad with 1,000 plaque-forming units (PFU) 6 h after treatment with phosphate-buffered saline or an isotype control IgG, most mice succumbed (became moribund, meeting euthanasia criteria) within 6 days (Fig. 4e). However, all mice treated with PCDH10_EC1_–Fc survived infection. Therefore, PCDH10_EC1_–Fc can protect mice against a highly virulent WEEV strain that can engage multiple receptors on brain cells.

## WEEV recognition of receptor orthologues

**Fig. 5:**
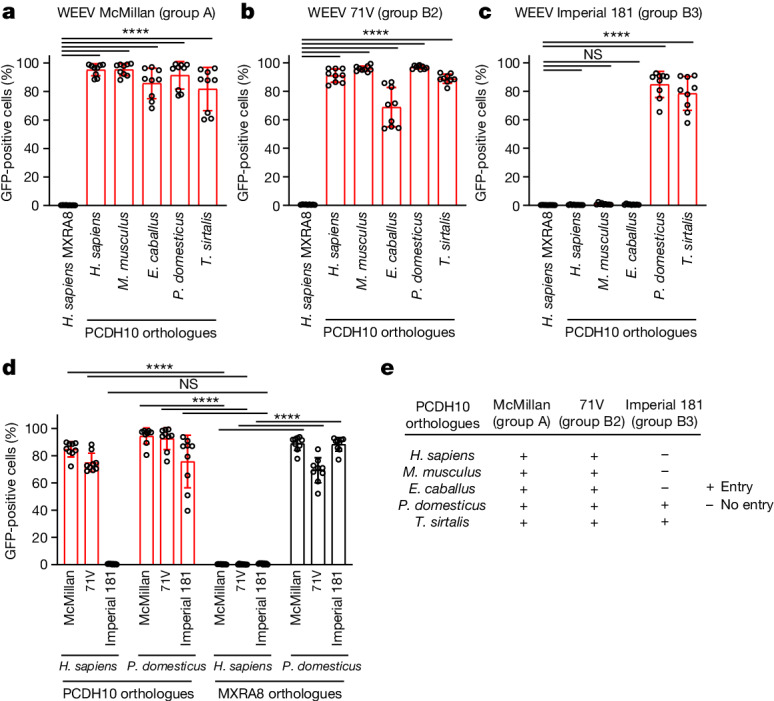
Species-specific recognition of PCDH10 is preserved during WEEV evolution. **a**–**c**, K562 cells stably expressing PCDH10 orthologues from humans (*Homo sapiens*), mice (*M. musculus*), horses (*E. caballus*), house sparrows (*P. domesticus*) and common garter snakes (*T. sirtalis*) were infected with GFP-expressing WEEV McMillan (**a**), 71V (**b**) or Imperial 181 (**c**) RVPs. Cells expressing human MXRA8 were used as controls. Infection was quantified by flow cytometry. **d**, K562 cells stably expressing human or house sparrow orthologues of PCDH10 or MXRA8 were infected with GFP-expressing WEEV RVPs of indicated strains. Infection was quantified by flow cytometry. **e**, Summary of infectivity assays on K562 cells stably expressing indicated orthologues of PCDH10. **a**–**d**, Data are mean ± s.d. from 3 experiments performed in triplicates (*n* = 9). One-way ANOVA with Dunnett’s multiple comparisons test (**a**–**c**); two-way ANOVA with Dunnett’s multiple comparisons test (**d**). Source Data

**Extended Data Fig. 10:**
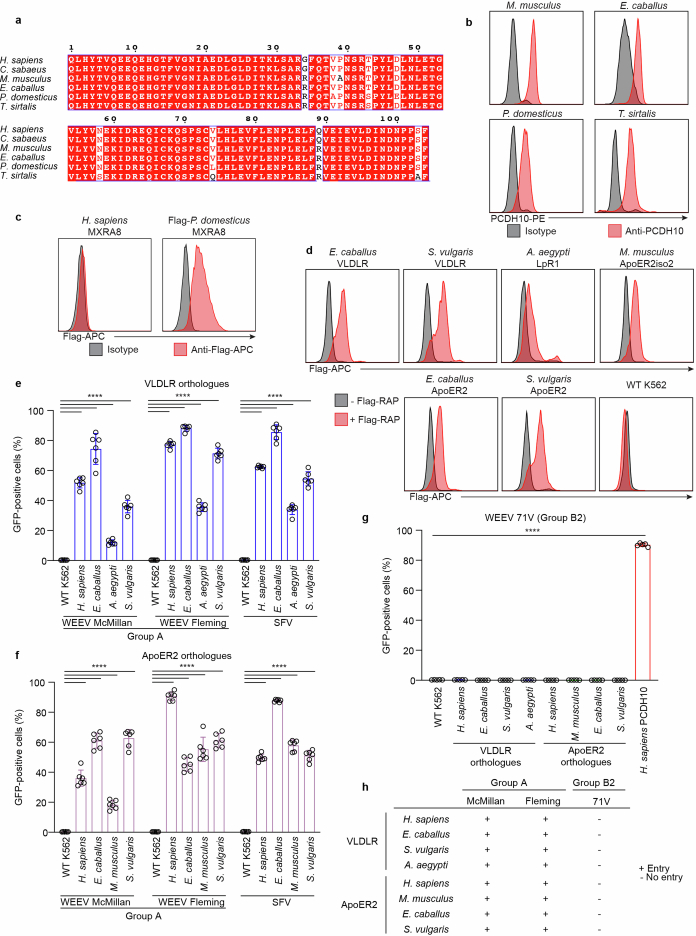
Orthologue compatibility of PCDH10, VLDLR, and ApoER2 with WEEV strains. **a**, Sequence alignment of PCDH10 EC1 from humans (*H. sapiens*), mice (*M. musculus*, GenBank: NP_001091640.1), horses (*E. caballus*, GenBank: XP_023492316.1), house sparrows (*P*. *domesticus*), and common garter snakes (*T. sirtalis*, GenBank: XP_013928164.1). The alignment was generated using Esprit 3.0^68^. **b**, Cells surface staining of K562 cells stably expressing PCDH10 orthologues of mice (*M. musculus*), horses (*E. caballus*), house sparrows (*P. domesticus*), and common garter snakes (*T. sirtalis*), as monitored by flow cytometry. **c**, Cell surface staining of K562 cells stably expressing human MXRA8 or Flag-tagged house sparrow MXRA8, as monitored by flow cytometry. **d**, Cell surface staining of K562 cells overexpressing VLDLR and ApoER2 orthologues from indicated species as monitored by flow cytometry. RAP-Flag was used as a surrogate for primary antibody and RAP staining was detected using an APC-conjugated anti-Flag antibody. **e**–**f**, Wild-type (WT) K562 cells or K562 cells stably expressing orthologues of VLDLR (**e**) or ApoER2 (**f**) were infected with GFP-expressing RVPs of WEEV strain McMillan, strain Fleming, or SFV. Infection was quantified by flow cytometry. **g**, WT K562 cells or K562 cells stably expressing orthologues of VLDLR or ApoER2, or human PCDH10, were infected with GFP-expressing WEEV 71 V RVPs. **h**, Summary of infectivity assays on K562 cells expressing orthologues of VLDLR and ApoER2. Data are mean ± s.d. from two experiments performed in triplicates (n = 6) (**e**, **f**), or one experiment performed in duplicates and one experiment performed in triplicates (n = 5) (**g**). Two-way ANOVA with Dunnett’s multiple comparisons test, ****P < 0.0001 (**e**, **f**). One-way ANOVA with Dunnett’s multiple comparisons test, ****P < 0.0001 (**g**). Source Data

WEEV infects nonhuman mammals, birds, reptiles and amphibians^3,10,39–42^. PCDH10 is highly conserved across vertebrate species, particularly in EC1 (Extended Data Fig. 10a), the presumed site of WEEV attachment. We tested the orthologues from horses (*Equus caballus*, epizootic spillover hosts), house sparrows (*Passer domesticus*, principal enzootic reservoir and amplification hosts), common garter snakes (*Thamnophis sirtalis*, proposed overwintering hosts^40,41^) and mice (*Mus musculus*, experimental model system^8^). Expression of all tested orthologues rendered K562 cells permissive to RVP infection by group A strain McMillan and group B2 strain 71V (Fig. 5a,b and Extended Data Fig. 10b).

Orthologues of VLDLR and ApoER2 from various species can support infection by EEEV, SFV and SINV to varying degrees, potentially explaining the wide host range of these alphaviruses^4^. We tested whether the group A WEEV strains could have engaged VLDLR and ApoER2 during infection of human, avian, equine and mosquito hosts. We found that RVPs of two group A WEEV strains, McMillan and Fleming, could infect K562 cells expressing the VLDLR orthologues of horses, starlings (*Sturnus vulgaris*) and mosquitoes (*Aedes aegypti*), as well as ApoER2 orthologues of mice, horses and starlings (Extended Data Fig. 10d–f). Group B2 WEEV strain 71V did not recognize any tested VLDLR or ApoER2 orthologues (Extended Data Fig. 10g,h).

## Imperial 181 strain binds avian PCDH10

Group B3 WEEV strain Imperial 181, isolated in 2005 from *Culex tarsalis* mosquitoes in Imperial County, California, causes no mortality in inoculated mice or Syrian hamsters^2^. This strain does not recognize human PCDH10, VLDLR, or ApoER2 (Fig. 3a,b). Because the Imperial 181 strain has similar fitness in house sparrows as the group B1 WEEV strain BFS932^3^, which binds human PCDH10 (Fig. 3a,b), we suspected that avian hosts express cellular receptors that Imperial 181 recognizes. Indeed, K562 cells transduced to express sparrow PCDH10 were rendered permissive to infection with Imperial 181 RVP strain (Fig. 5c). K562 cells expressing common garter snake PCDH10 were similarly rendered permissive to WEEV Imperial 181 RVPs (Fig. 5c), suggesting that recognition of reptilian PCDH10 also has been preserved in this strain.

Whereas mammalian orthologues of MXRA8 are receptors for CHIKV and some other alphaviruses^27^, a recent study found that avian orthologues of MXRA8 can serve as receptors for WEEV, SINV and other WEE complex alphaviruses with avian reservoirs^43^. That study examined only WEEV E2–E1 spike protein sequences for CBA87 (South America) and McMillan (group A) strains. We found that overexpression of sparrow MXRA8, but not human MXRA8, could mediate entry of WEEV Imperial 181 RVPs and 71V (group B2) strain RVPs (Fig. 5d and Extended Data Fig. 10c), suggesting that recognition of avian MXRA8 has similarly been preserved.

## Discussion

**Extended Data Fig. 11:**
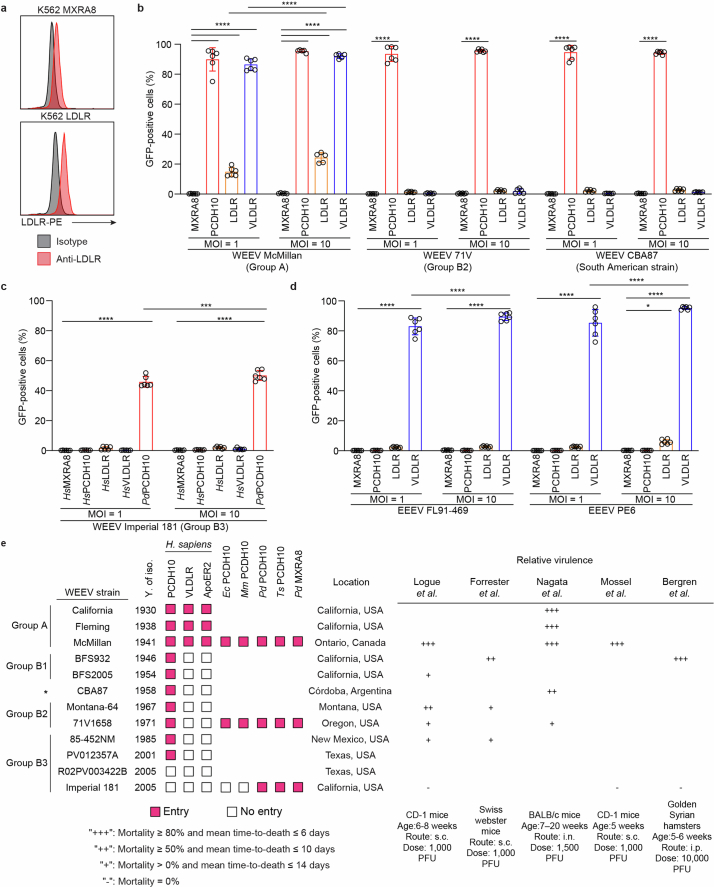
Assessment of WEEV strain LDLR-dependency and summary of receptor dependencies. **a**, Cell surface staining for LDLR on K562 cells overexpressing human MXRA8 or LDLR, as monitored by flow cytometry. **b**–**d**, K562 cells overexpressing human MXRA8, PCDH10, LDLR, or VLDLR were infected with GFP-expressing RVPs for WEEV McMillan, CBA87, 71 V (**b**), Imperial 181 (**c**), or EEEV strains FL91-469 or PE6 (**d**). K562 cells overexpressing house sparrow (*P. domesticus*) PCDH10 were used as a positive control in infectivity assay with WEEV Imperial 181 RVPs (**c**). **e**, Summary of strains tested in this study, including phylogenetic groups, year/location of isolation, human receptor compatibility, and relative virulence in mammalian models. “*” indicates South American origin of strain CBA87, which is phylogenetically distinct from North American groups. Information on mammalian virulence was summarized from studies by Logue et al.^2^, Forrester et al.^69^, Nagata et al.^11^, Mossel et al.^18^, and Bergren et al.^3^. While we provide an estimate of virulence based on mortality and mean time-to-death, specific animal models and experimental designs are also included here for each study as these differences may affect interpretation of virulence and may not be readily standardizable. s.c., subcutaneous; i.n., intranasal; i.p., intraperitoneal. Data are mean ± s.d. from two experiments performed in triplicates (n = 6) (**b**, **c**, **d**), except for WEEV McMillan (**b**), for which one experiment was performed in duplicates and on experiment was performed in triplicates (n = 5). Two-way ANOVA with Šídák’s multiple comparisons test, *P = 0.0113, ***P = 0.0005, ****P < 0.0001 (**b**, **c**, **d**). Source Data

WEEV submergence has been marked by a decline in mammalian virulence^2,3^ (Extended Data Fig. 11e), but the precise molecular drivers remain unknown. Here we show that PCDH10 is a general receptor for WEEV, but a WEEV strain that was isolated recently in North America has lost the ability to recognize human PCDH10 but retains the ability to bind avian and reptilian PCDH10. Although PCDH10 is not structurally related to the LDLR-related receptors that are implicated in the entry of multiple alphaviruses^4,26^, PCDH1 (a δ1-protocadherin expressed in the lung) is a receptor for certain hantaviruses that cause pulmonary syndromes^44^. Thus, tissue distribution of protocadherins may direct tissue-specific tropism and pathogenesis of viruses that recognize this family of proteins as receptors. We tested individual strains of EEEV, VEEV, SFV, SINV and CHIKV, but cannot exclude that other strains of these alphaviruses may bind PCDH10.

On the basis of available sequences, we found pathogenic ancestral WEEV strains that caused large-scale outbreaks in the 1930s and 1940s could recognize PCDH10, VLDLR and ApoER2. Of note, EEEV, an alphavirus that continues to cause outbreaks, also binds VLDLR and ApoER2^4^. Source group A WEEV sequences that we used to generate RVPs or rescue molecular clones were derived from isolates that had been serially passaged in suckling mouse brains^1,45^, a common practice in the early twentieth century. It is possible that some group A strains acquired the ability to bind LDLR-related proteins during this passaging. However, additional strains we used that only bind human PCDH10 had been similarly passaged, albeit less extensively (Supplementary Table 1). Nonetheless, our experiments suggest that the ability to acquire the use of VLDLR and ApoER2 as cellular receptors is likely to be an intrinsic, ancestral property of the WEEV spike protein that should be considered during risk assessment of emerging strains and in the development of countermeasures.

The inability to bind mammalian PCDH10, VLDLR or ApoER2 may be associated with decreased virulence in mouse models of recent WEEV strains compared with ancestral strains^2,3,18^. A study showed that three strains that we found to recognize PCDH10 only (BFS2005, 71V and 85-452NM) do not have significantly different virulence, despite being isolated over a period of three decades^2^ (Extended Data Fig. 11e). Strains of distinct lineages (B1, B2, B3 and South American) that recognize PCDH10 but not LDLR-related receptors have been found to be similarly virulent in mice^2,11^ (Extended Data Fig. 11e). These studies and our findings suggest that mammalian virulence of WEEV is associated with receptor usage patterns and not necessarily time of isolation or phylogenetic relationship.

A recent study using SFV suggests that binding to VLDLR and ApoER2 is important for neuroinvasion in mice^46^. Determining how the ability to bind mammalian PCDH10 versus LDLR-family members influences WEEV strain virulence would require additional experiments, including in vivo studies with strains that contain E2 and E1 spike protein mutations that selectively ablate the ability to bind PCDH10 or LDLR-related proteins.

Notably, the group B3 Imperial 181 strain (2005), which does not bind mammalian PCDH10, and group B1 BFS932 (1946), which does, were found to be equally fit in competition assays performed in house sparrows^3^.The ability to recognize avian orthologues of PCDH10 and MXRA8 potentially provides a mechanism for WEEV to maintain fitness in its avian reservoir upon losing the ability to bind mammalian receptors. Additional studies will be required to clarify the relative roles of WEEV binding to PCDH10 or MXRA8 in infection of avian hosts.

Neither Imperial 181 (California) nor R02PV003422B, another group B3 strain (Texas), binds human PCDH10, suggesting that this phenotypic variant has been geographically dispersed. Whether strains that cannot bind human PCDH10 have become dominant in North America awaits further environmental sampling and surveillance efforts. Of note, because fitness of WEEV for transmission by mosquito vectors among avian reservoir hosts has not significantly decreased^3^, strains that can recognize human PCDH10 as a receptor may be poised for re-emergence.

We also found that the Imperial 181 and R02PV003422B strains could infect Vero E6 cells (Extended Data Fig. 7b). The EC1 domains of African green monkey (*Chlorocebus sabaeus*) and human PCDH10 are identical (Extended Data Fig. 10a), suggesting that Imperial 181 and R02PV003422B strains are also unlikely to bind *C. sabaeus* PCDH10. PCDH10 polyclonal antibodies, which bind Vero E6 cells, and the LDLR-related receptor antagonist RAP, had little effect on infection of this cell type by McMillan, 71V or Imperial 181 RVPs (Extended Data Fig. 7c,d). Therefore, WEEV is likely to be able to enter Vero cells using a pathway that is independent from VLDLR, ApoER2 or PCDH10.

It is unclear whether the lack of binding of contemporary WEEV strains to mammalian orthologues of PCDH10 has occurred through genetic drift or as a result of evolutionary pressure. Over the course of its evolution, WEEV may have had decreasing ecological opportunities for epizootic circulation because of the industrialization of agriculture, fewer horses on farmlands, and vaccination in horses^3^. Because WEEV could be maintained in enzootic circulation independent of epizootic hosts, recognition of receptor orthologues in epizootic hosts no longer conferred an evolutionary advantage, if there was any (for example, through a potential role of equids as amplification hosts)^3,47,48^, and became susceptible to elimination owing to drift.

LDLR was recently implicated as a receptor for multiple alphaviruses, and as a low affinity receptor for WEEV (strain CBA87) and EEEV^49,50^. We tested whether WEEV McMillan, a group A strain that binds VLDLR and ApoER2, could also bind LDLR. We found that LDLR overexpression could enhance entry of WEEV McMillan RVPs, albeit to limited extents (Extended Data Fig. 11a,b), suggesting that McMillan, and possibly other group A WEEV strains, may engage LDLR with low affinity to promote viral entry into neuronal cell types that express this receptor. This observation is consistent with our observation that McMillan RVPs exhibited some residual entry in *Pcdh10*^−/−^ mouse neurons treated with RAP (Fig. 4a,b), a protein that antagonizes VLDLR and ApoER2 but not LDLR itself when added exogenously^51–54^. However, we observed little to no effects of ectopic LDLR expression on WEEV CBA87 (South American strain), 71V (group B2) or Imperial 181 (group B3) RVPs, or RVPs for two EEEV strains (Extended Data Fig. 11b–d). Differences in multiplicity of infection or systems used to study viral entry (for example, single-cycle RVPs instead of replication-competent chimeric SINV) may account for these differences.

Considering the recent outbreak in South America, our results may provide guidance for estimating the threat of re-emerging strains. We demonstrated that South American strain WEEV CBA87 (Argentina, 1958) binds human PCDH10, but not VLDLR or ApoER2, suggesting that human PCDH10 is a receptor for WEEV strains that have circulated in South America.

Several contemporary WEEV strains have lost the ability to bind multiple mammalian receptors over the course of WEEV evolution, providing a potential molecular basis for decreased mammalian virulence accompanying the decrease in epizootic activity of a major encephalitic arbovirus in North America. We propose that the inability of contemporary North American strains to recognize mammalian orthologues of PCDH10 is a molecular hallmark of the epizootic decline of WEEV. Outbreak preparedness could be bolstered by future studies to identify the key sequence polymorphisms in the WEEV spike E2 and E1 proteins that explain shifted receptor compatibilities.

## Methods

### Cells and viruses

HEK 293T (human kidney epithelial, ATCC CRL-11268), Vero E6 (*Cercopithecus aethiops* kidney epithelial, ATCC CRL-1586) and SVG-A (human astroglial, provided by T. Kirchhausen) cells were maintained in Dulbecco’s modified Eagle’s medium (DMEM, Gibco) supplemented with 10% (v/v) fetal bovine serum (FBS) and 25 mM HEPES (Thermo Fisher Scientific). Vero 81 (*C. aethiops* kidney epithelial, ATCC CCL-81) cells were cultured in DMEM high glucose supplemented with 10% (v/v) FBS, 1% (v/v) penicillin-streptomycin, 1× non-essential amino acids (NEAA, Sigma), and 1 mM sodium pyruvate. K562 (human chronic myelogenous leukaemia, ATCC CCL-243) cells were maintained in RPMI1640 (Thermo Fisher Scientific) supplemented with 10% (v/v) FBS, 25 mM HEPES, and 1% (v/v) penicillin-streptomycin. SK-N-SH (human brain neuroblastoma, ATCC HTB-11) cells were maintained in Eagle’s minimum essential medium (EMEM, Sigma) supplemented with 10% (v/v) FBS, 25 mM HEPES, and 1% (v/v) penicillin-streptomycin. Expi293F cells (Thermo Fisher Scientific A14527) were maintained in Expi293 Expression Medium (Thermo Fisher Scientific). Cell lines were not authenticated. Absence of mycoplasma is confirmed through routine mycoplasma test using e-Myco PCR detection kit (Bulldog Bio 25234).

Full-length infectious clones of WEEV Fleming and WEEV McMillan have been previously described^55^ and were provided by W. Klimstra. Plasmids were transformed into TOP10 *Escherichia coli* (Invitrogen) and prepared using the Plasmid Plus Midi or Maxi kits according to the manufacturer’s protocol (Qiagen). Linearization of 10 µg of plasmid was achieved with the NotI-HF restriction enzyme (NEB), followed by phenol-chloroform extraction. WEEV RNA was in vitro transcribed using the mMESSAGE mMACHINE T7 kit (Invitrogen) with 1 µg linearized plasmid. Following RNA transcription, two confluent T-150 or T-175 flasks of Vero 81 (for Fleming) or Vero E6 (for McMillan) cells were detached with 0.25% trypsin-EDTA (Gibco) and washed three times with Dulbecco’s phosphate-buffered saline (DPBS). Following the final wash, cells were resuspended in DPBS and combined with the entire volume of transcribed RNA in a 4 mm gap cuvette. The cells and RNA were subjected to three 250 V, 10 ms pulses at 1 s intervals in an ECM 830 square wave electroporation system (BTX). Cells were allowed to rest for approximately 10 min at room temperature before being transferred to a T-75 flask in the presence of medium with reduced FBS and maintained at 37 °C with 5% CO_2_. Upon onset of cytopathic effect two days post-electroporation, cellular debris was pelleted by centrifugation and viral stocks were collected and stored at −80 °C.

### Primary mouse cortical neuron culture and infection

Mouse experiments were approved under the Harvard Medical School Institutional Animal Care and Use Committee (protocol number IS00002530-3), and under the Boston Children’s Hospital Institutional Animal Care and Use Committee (protocol number 00001725). The *Pcdh10*-knockout mouse line was maintained on a C57BL/6J background^21^. Postnatal day 0 or day 1 pups were genotyped by genomic PCR, in which a fragment of the wild-type allele was amplified by primer P1 (5′-GCTCGCGTTTGCCAGCCGTTGATATC-3′) and primer P2 (5′- AGAGCGTCTCCAAATCGAGCCTCATT-3′), and a fragment of the mutant (null) allele was amplified by primer P1 and primer P3 (5′-ACTGGTACACGCGACTGAAAACAGTG-3′). Cortical neurons were dissected and dissociated from postnatal day 1 or 2 neonates using methods adapted from ref. ^56^. In brief, pups were anaesthetized on ice and euthanized by decapitation. The cortices were then isolated in cold HBSS and dissociated in HBSS supplemented with 20 units ml^−1^ of papain (Worthington Biochemicals) and 2000 units ml^−1^ of DNase I (Roche). During dissociation, the cortices were first incubated at 37 °C for 5 min following trituration. Following dissociation, the papain was neutralized with 10 mg ml^−1^ ovomucoid inhibitor (Worthington Biochemicals) in HBSS. Cells were then washed once with neurobasal medium by centrifugation at 600*g* for 3 min and plated at a density of 100,000 cells per well in 96-well plates (Cellvis) coated with 20 μg ml^−1^ poly-l-lysine (Sigma) and 4 μg ml^−1^ laminin (Thermo Fisher). The neurons were maintained in neurobasal medium supplemented with B27 (Thermo Fisher), l-glutamine and penicillin-streptomycin, unless specified otherwise. The plated neurons were treated with 3 µM cytosine arabinoside (AraC) from day 1 post-plating (day in vitro 1 (DIV 1)) to DIV 3 to reduce non-neuronal cell outgrowth. On DIV 4 we pre-incubated WEEV or SFV RVPs with 100 µg ml^−1^ transferrin or RAP in culture medium containing 5 µg ml^−1^ polybrene for 30 min at 37 °C. We then added the mixtures to cells. Cells were imaged every 4 h for 24 h using the Incucyte S3 Live Cell Imaging system (Sartorius) with Incucyte S3 Software version 2022B Rev2 (Sartorius) using a 20× objective. GFP-positive neurons were scored as cells with a threshold signal greater than 5 green calibrated units (GCU) above background, using a Top-hat background subtraction method. The neuronal cell body area in each image was obtained by analysing phase-contrast images using the Incucyte S3 Software. To calculate the percentage of positive cells, at the time point of 24 h post-infection, the area of GFP signal above background was divided by the total area covered by neuronal cell bodies and was multiplied by 100. We calculated relative infection as follows: Relative infection (%) for wild-type neurons = (percentage of GFP-positive wild-type cells in the presence of transferrin or RAP)/(percentage of GFP-positive wild-type cells in the absence of transferrin or RAP) × 100; relative infection (%) for *Pcdh10*^−/−^ neurons = (percentage GFP-positive *Pcdh10*^−/−^ cells in the presence or absence of transferrin or RAP)/(percentage GFP-positive wild-type cells in the absence of transferrin or RAP) × 100.

### Reporter virus particle generation

RVPs were generated as previously described^4^. In brief, we transfected two plasmids into HEK 293T cells using Lipofectamine 3000 (Thermo Fisher): a modified pRR64 Ross River virus replicon^57^ provided by R. Kuhn (Purdue University) (the SP6 promoter is replaced with a CMV promoter, the E3–E2–(6 K/TF)–E1 sequence is replaced with a turbo GFP or CD20 reporter preceded by a porcine teschovirus-1 2 A self-cleaving peptide), and a pCAGGS vector expressing heterologous alphavirus E3–E2–(6 K/TF)–E1 proteins. At 4–6 h post-transfection, we replaced medium with Opti-MEM (Thermo Fisher) supplemented with 5% (v/v) FBS, 25 mM HEPES, and 5 mM sodium butyrate. We collected supernatant 2 days post-transfection, centrifuged supernatant at 4,000 rpm for 5 min, filtered these using a 0.45-µm filter, and froze aliquots at −80 °C for storage.

Alphavirus E3–E2–(6 K/TF)–E1 coding sequences cloned into the pCAGGS vector include: WEEV strain 71V1658 (GenBank NC_003908.1), WEEV strain California (GenBank KJ554965.1), WEEV strain Fleming (GenBank MN477208.1), WEEV strain McMillan (GenBank GQ287640.1), WEEV strain BFS932 (GenBank KJ554966.1), WEEV strain BFS2005 (GenBank GQ287644.1), WEEV strain Y62-33 (GenBank KT844544.1), WEEV strain Montana-64 (GenBank GQ287643.1), WEEV strain CU71-CPA (GenBank KT844545.1), WEEV strain 85-452NM (GenBank GQ287647.1), WEEV strain PV012357A (GenBank KJ554987.1), WEEV strain R02PV003422B (GenBank KJ554990.1), WEEV strain Imperial 181 (GenBank GQ287641.1), SFV strain SFV4 (GenBank AKC01668.1), EEEV strain Florida 91-469 (GenBank Q4QXJ7.1), EEEV strain PE6 (GenBank AY722102.1), SINV strain Toto1101 T6P144 (GenBank AKZ17594.1), VEEV strain INH-9813 (GenBank KP282671.1) and CHIKV strain 37997 (GenBank AY726732.1).

### Reporter virus particle titration

Titration of GFP-expressing RVPs was performed on Vero E6 cells seeded in 96-well plates using a serial twofold or tenfold dilution of the RVP stocks. At 24 h post-infection, numbers of GFP-positive cells were counted using fluorescence microscopy and used to calculate RVP titre as infectious unit per millilitre (IU ml^−1^), assuming that at high dilution factors, 1 GFP-positive cell = 1 infectious unit, given that RVPs can only infect cells for one cycle.

### sgRNA library design, screening, analysis

Supplementary Table 2 contains genes targeted in the single guide RNA (sgRNA) library. The library, as previously described^4^, includes genes that encode proteins identified by mass spectrometry to be on the cell surface^58^ and proteins either bioinformatically predicted to be on the cell surface^59,60^ or annotated as associated with endosomes, lysosomes, vesicles or the cell surface by UniProt (https://www.uniprot.org). We cloned the guide RNAs into lentiGuide-Puro^61^ (provided by F. Zhang, Addgene #52963) and amplified the library in Endura ElectroCompetent cells (Lucigen 60242) as previously described^62^. We packaged the library into lentivirus by transfecting the plasmid library along with pMD2.G (provided by D. Trono, Addgene #12259) and psPAX2 (provided by D. Trono, Addgene #12260) into HEK 293T cells using Lipofectamine 3000 (Thermo Fisher). Supernatants were collected 1 and 2 days post-transfection, pooled, clarified by centrifugation (1,200 rpm for 5 min), filtered through a 0.45-µm membrane, and stored at −80 °C.

We used a previously described HEK 293T line that stably expresses *Streptococcus pyogenes* Cas9 (HEK 293T-Cas9) for the CRISPR–Cas9 screen^4^. We transduced HEK 293T-Cas9 cells with the CRISPR sgRNA lentivirus library at a MOI of 0.3. One day post-transduction, we began selection of sgRNA expressing cells by adding puromycin at 1 µg ml^−1^. Seven to ten days post-selection, we infected cells with WEEV RVPs expressing CD20 (strain 71V1658), aiming for 80–90% infected cells compared to HEK 293T-Cas9 cells not transduced with the library, as monitored by an anti-CD20 APC-conjugated antibody (Miltenyi Biotec Clone LT20 130-113-370) used at 1:50 dilution. Three days post RVP infection, we depleted infected cells using anti-CD20 MicroBeads (Miltenyi Biotec 130-091-104). To improve the signal-to-noise ratio, we performed two additional rounds of infection with WEEV RVPs expressing CD20 following expansion of uninfected cells. We extracted genomic DNA from uninfected cells and library-transduced HEK 293T-Cas9 cells that had not been infected with RVPs. We amplified sgRNA sequences and determined sgRNA content using next-generation sequencing on an Illumina MiSeq. Tag sequences were removed and gene enrichment was analysed using MAGeCK (version 0.5.6)^20^.

### Genetic knockout and validation

We used previously described VLDLR-knockout HEK 293T cells^4^. To disrupt *PCDH10* using CRISPR–Cas9, we transduced HEK 293T cells with pairs of sgRNAs targeting two sites into lentiGuide-Puro (Addgene #52963) along with lentiCas9-blast (Addgene #52962). We then isolated individual clones using clonal dilution. We confirmed the lack of cell surface expression of PCDH10 or VLDLR in knockout clones by staining cells with an anti-PCDH10 antibody (Proteintech 21859-1-AP) or an anti-VLDLR antibody (GeneTex GTX79552). Clonal PCDH10-knockout cells were genotyped by PCR.

Forward guide RNA sequences used to disrupt *PCDH10* were: sg*PCDH10*-1, 5′-CGTGACTGACCGCGACTCAG-3′; sg*PCDH10*-2, 5′-TCGCATGGACTGGCGCACCG-3′. Genotyping primer sequences for clonal PCDH10-knockout HEK 293T cells are: forward, 5′-CTACACGGTACAGGAGGAGC-3′; reverse, 5′-CCAACGCGATGATGAGGATG-3′.

### Expression and purification of VLPs

We transfected plasmids encoding the structural polyprotein (capsid–E3–E2–(6 K/TF)–E1) of CHIKV strain 37997^63^, WEEV strain CBA87, which contains an nuclear-localization mutation in the gene encoding the capsid protein^29^, or WEEV strain McMillan (GenBank GQ287640.1) containing the same mutation into Expi 293F cells using the ExpiFectamine 293 Transfection Kit (Thermo Fisher) according to the manufacturer’s protocol. Culture supernatant was collected 5 days post-transfection and cleared of cell debris by centrifugation at 3,000*g* for 20 min. The clarified supernatant was laid upon 5 ml 35% (w/v) sucrose cushion on top of 5 ml 70% (w/v) sucrose cushion, and ultracentrifuged at 25,000 rpm for 5 h at 4 °C. VLPs were pooled from the interface of the 35% and 70% sucrose cushion and buffer exchanged in a 100-kDa Amicon filter (Sigma) to lower the sucrose concentration to less than 20% at a volume of 1 ml. We then laid VLPs onto a 20%–70% continuous sucrose density gradient and ultracentrifuged samples at 35,000 rpm for 1.5 h at 4 °C. The VLP band was collected. VLPs were stored at 4 °C without buffer exchange and not frozen. We confirmed particle integrity and the absence of degradation products using SDS–PAGE. VLPs were always used within seven days of purification, and buffer exchanged based on the application immediately before use.

### Fluorescent labelling of VLPs

Purified VLPs were buffer exchanged into 0.1 M sodium bicarbonate (pH 8.3) and diluted to a concentration of 1 mg ml^−1^. Immediately before use, Alexa Fluor 647 (AF647) NHS ester (succinimidyl ester) (Invitrogen A37573) was dissolved in dimethyl sulfoxide (DMSO) at a final concentration of 1 mg ml^−1^. We added 25 µg of AF647 NHS ester to 1 mg of VLP and incubated the mixture for 30 min at room temperature. We removed excess dye from the solution with a Zeba Spin Desalting Column (Thermo Fisher) and buffer exchanged labelled VLPs into PBS. Labelled VLPs were stored at 4 °C and used for confocal microscopy experiments within 12 h of labelling.

### Ectopic expression construct design and generation of stable cell lines

cDNA encoding human PCDH10 (GenBank NM_032961.3), mouse PCDH10 (GenBank NM_001098170.1), human VLDLR (GenBank NP_003374.3), human MXRA8 (GenBank NM_032348.3), and human LDLR (GenBank AAP88892) were obtained from GeneScript. The coding sequences of *P. domesticus* PCDH10 and *P. domesticus* MXRA8 were obtained by aligning the coding sequences of *Passer montanus* PCDH10 (GenBank XM_039733439.1) and *P. montanus* MXRA8 (GenBank XM_039727729.1) against the genome of *P. domesticus* (GenBank GCA_001700915.1) and assembling aligned fragments. Gene blocks were synthesized at Integrated DNA technologies (IDT) for the following codon-optimized coding sequences: *E. caballus* PCDH10 (GenBank XM_023636548.1), *P. domesticus* PCDH10, *P. domesticus* MXRA8, *T. sirtalis* PCDH10 (GenBank XM_014072689.1), *H. sapiens* ApoER2 isoform 2 (GenBank NM_004631.5). A Flag tag (DYKDDDDK) was placed at the N-terminus of *P. domesticus* MXRA8 to monitor expression. Truncation constructs of human PCDH10 were generated as follows: PCDH10(ΔEC1) was generated by removing EC1 (Q19–F122) in the PCDH10 precursor protein sequence (numbering includes the signal peptide sequence); PCDH10 stalk–Flag was generated by removing Q19–G690 in the precursor protein sequence and adding a Flag tag between S696 and G697; PCDH10 EC1–Flag and EC2–Flag were generated by replacing Q19–G690 in the precursor protein sequence with EC1 (Q19–F122) or EC2 (P123–F250) and inserting a Flag tag between S696 and G697; PCDH10(ΔCT) was generated by removing the cytoplasmic domain (Q741–C1040 in the precursor protein sequence) of PCDH10. PCDH10–GPI and VLDLR–GPI were generated by replacing the transmembrane helices and cytoplasmic domains of PCDH10 (L716–C1040) and VLDLR (A798–A873) with a GPI anchor coding sequence (5′-CCTAATAAGGGCTCAGGCACTACTTCAGGAACCACCAGACTGCTGTCTGGCCATACCTGCTTTACACTGACCGGTCTCCTGGGGACGCTGGTCACCATGGGACTGCTGACC-3′), which encodes a GPI anchor peptide (PNKGSGTTSGTTRLLSGHTCFTLTGLLGTLVTMGLLT).

The above constructs were cloned into lentiGuide-Puro (Addgene #52963). We transfected this vector along with psPAX2 (Addgene #12260) and PMD2.G (Addgene #12259) at a ratio of 3:2:1 into HEK 293T cells using Lipofectamine 3000 (Thermo Fisher). Lentiviruses were collected 2 days post-transfection and used to transduce K562 cells or clonal *PCDH10*-knockout HEK 293T cells. Successfully transduced K562 cells and HEK 293T cells were selected using puromycin at 2 μg ml^−1^ and 1 μg ml^−1^ respectively. K562 cells transduced with *E. caballus* PCDH10 were additionally sorted using fluorescence-activated cell sorting with anti-PCDH10 polyclonal antibodies (Proteintech 21859-1-AP) to isolate a sub-population of positive cells. Cell lines were confirmed to express the transduced constructs by cell surface antibody staining.

### Cell surface antibody staining

Primary antibodies were diluted to 10 μg ml^−1^ in binding buffer (2% (v/v) goat serum in PBS) immediately before use. Primary antibodies used include polyclonal anti-PCDH10 (Proteintech 21859-1-AP), anti-VLDLR (GeneTex GTX79552), anti-MXRA8 (MBL International W040-3), anti-LDLR (R&D Systems MAB2148), rabbit IgG isotype (Proteintech 30000-0-AP) and mouse IgG isotype (BD Biosciences BDB557351). Cells were incubated in blocking buffer (5% (v/v) goat serum in PBS) for 30 min at 4 °C followed by incubation with primary antibodies at 10 µg ml^−1^ in binding buffer (2% (v/v) goat serum in PBS). Cells were washed three times in binding buffer and subsequently incubated with a PE-conjugated donkey anti-rabbit F(ab′)_2_ fragment (Jackson ImmunoResearch 711-116-152) or a PE-conjugated donkey anti-mouse F(ab′)_2_ fragment (Jackson ImmunoResearch 715-116-150) diluted 1:200 in binding buffer for 30 min at 4 °C. We washed cells twice in binding buffer and twice in PBS, fixed cells in 2% (v/v) formalin and detected cell surface receptor expression using an iQue3 Screener PLUS (Intellicyt) with ForeCyt (Sartorius) software. Antibody staining was visualized using FlowJo (version 10.6.2).

For cells expressing Flag-tagged constructs, we diluted an APC-conjugated anti-DYKDDDDK (Flag) antibody (BioLegend 637307) or an APC-conjugated control antibody (BioLegend 402306) to 5 μg ml^−1^ in binding buffer immediately before use. Cells were blocked as described above, incubated with primary antibodies in binding buffer for 30 min at 4 °C, and washed twice in binding buffer, twice in PBS. We then detected cell surface receptor expression using an iQue3 Screener PLUS (Intellicyt) with IntelliCyt ForeCyt Standard Edition version 8.1.7524 (Sartorius) software. Antibody staining was visualized using FlowJo (version 10.6.2).

### Protein purification

We cloned human PCDH10 EC1 (Q19–F122, GenBank NP_116586.1), human MXRA8 ectodomain (V20–H337, GenBank NP_001269511.1), human VLDLR ligand-binding domain (A31–C355, GenBank NP_003374.3) into a pVRC expression vector encoding the human IgG1 Fc as a fusion protein at the C-terminus, provided by A. Schmidt^64^. We cloned full-length human RAP (residues 1–353, including the signal sequence) (GenBank NP_002328) into the pCAGGs vector.

To produce PCDH10_EC1_–Fc and MXRA8_ect_–Fc, we transfected the pVRC vectors into Expi 293F cells using the ExpiFectamine 293 Transfection Kit (Thermo Fisher) according to the manufacturer’s recommendations. At 5 days post-transfection, we purified Fc fusion proteins using the MabSelect SuRe LX protein A affinity resin (GE Healthcare) according to the manufacturer’s protocol and further by size-exclusion chromatography using a Superdex 200 increase column. Control IgG (C1A-H12 anti-SARS-CoV-2 spike antibody^65^) was similarly generated. Proteins were stored in PBS. Proteins used for in vivo experiments were not subjected to size exclusion chromatography other than for a small aliquot analyzed for quality control purposes; they were also tested for the presence of endotoxin, which was measured as <0.5 endotoxin units ml^–1^ using a Pierce Chromogenic Endotoxin Quantification Kit (Thermo Fisher Scientific).

To produce VLDLR_LBD_–Fc and RAP, we transfected the pVRC vector encoding VLDLR_LBD_–Fc and the pCAGGS vector encoding RAP into Expi 293F cells using the ExpiFectamine 293 Transfection Kit (Thermo Fisher) at a 1:1 ratio. 5 days post-transfection, culture supernatants were subjected to protein A affinity chromatography, during which VLDLR_LBD_–Fc bound by RAP was captured by the resin. We washed the resin with Tris-Buffered Saline (TBS) (20 mM Tris, 150 mM NaCl in water, pH 7.5) then eluted RAP with 300 column volumes of 10 mM EDTA in TBS overnight, then buffer exchanged RAP into TBS for storage. VLDLR_LBD_–Fc was refolded on the column by washing the resin with 100 column volumes of TBS containing 2 mM CaCl_2_ and subsequently eluted according to the manufacturer’s protocol. Proteins were further purified by size-exclusion chromatography using a Superdex 200 increase column. VLDLR_LBD_–Fc was buffered exchange into TBS containing 2 mM CaCl_2_ for storage.

### Inhibition of RVP entry by recombinant proteins or antibodies

We pre-incubated GFP-expressing alphavirus RVPs in the presence of recombinant proteins or antibodies and 5 µg ml^−1^ polybrene in culture medium for 30 min at 37 °C. The anti-PCDH10 antibodies and the control antibodies (anti-HLA-C polyclonal antibodies (Proteintech 15777-1-AP) and anti-HLA-ABC polyclonal antibodies (Proteintech 15240-1-AP) were first dialysed into PBS to remove azide preservatives. The mixtures were added to cells. 24 h post-infection, cells were washed twice in PBS and fixed in 2% (v/v) formalin. RVP entry was measured using an iQue3 Screener PLUS (Intellicyt) with IntelliCyt ForeCyt Standard Edition version 8.1.7524 (Sartorius) software. An example of the flow cytometry gating scheme used to quantify GFP expression after RVP infection is provided in Extended Data Fig. 2b. We calculated relative infection as follows: Relative infection (%) = (percentage of GFP-positive cells in the presence of recombinant proteins or antibodies)/(percentage of GFP-positive cells in the absence of recombinant proteins or antibodies) × 100.

### Confocal microscopy with fluorescently labelled VLPs

A total of 10^7^ K562 cells stably expressing human PCDH10 or MXRA8 were centrifuged at 1,000 rpm for 5 min and resuspended in a mixture of heparinases (heparinase I (R&D Systems 7897-GH) at 2 units ml^−1^, heparinase II (Sigma H8891) at 1 unit ml^−1^, heparinase III (R&D Systems 6145-GH) at 2 units ml^−1^). Cells were treated for 1 h at 37 °C. We then washed cells and resuspended cells to a density of 0.5 × 10^6^ ml^−1^ in RPMI1640 supplemented with 2% (v/v) FBS and 25 mM HEPES. Twenty-five micrograms of labelled VLPs were added to 0.5 × 10^6^ cells. For cells kept at 4 °C, cells were incubated on ice following addition of VLPs for 30 min. Cells were then washed once with cold PBS and kept on ice. Immediately before imaging, cells were treated with 500 µl Alexa Fluor 488-conjugated wheat germ agglutinin (WGA-AF488) (Invitrogen W11261) at 1 µg ml^−1^ in PBS, washed again with cold PBS, resuspended in 80 µl cold PBS, and placed in glass bottom microwell dishes (MatTek P35G-1.5-14-C) for imaging. Cells were imaged with a Yokogawa CSU-W1 single disk (50 µm pinhole size) spinning disk confocal unit attached to a Nikon Ti2 inverted microscope equipped with a Nikon linear-encoded motorized stage, an Andor Zyla 4.2 plus (6.5 µm photodiode size) sCMOS camera using a Nikon Plan Apo λS SR HP 100×C/1.45 Silicon DIC silicone immersion objective with Nikon Silicone oil. The final digital resolution of the image was 0.065 µm per pixel. Fluorescence from WGA-AF488 and VLPs conjugated to AF647 was collected by illuminating the sample with a solid state directly modulated 488 nm diode 100 mW (at the fibre tip) laser line or a solid state, directly modulated 640 nm diode 100 mW (laser tip) laser line in a Nikon LUNF XLlaser combiner, respectively. A hard-coated Semrock Di01-T405/488/568/647 multi-bandpass dichroic mirror was used for both channels. Signal from each channel was acquired sequentially with either a hard-coated Chroma ET525/36 m or Chroma ET700/75 m emission filters in a filter wheel placed within the scan unit, respectively. *z*-stacks were set by determining the top and bottom of the cell, using WGA-AF488 fluorescence as a reference. The approximate volume was ~20 µm, and the step size was set to 0.2 µm (approximating 80 *z*-slices in total), using the piezo stage insert (Mad City Labs, 500 µm). The exposure times were optimized to fill ~1/2–2/3 of the camera dynamic range and then kept consistent for one image set (individual exposure times are found in the image metadata and image legend where applicable). Fluorescence from each fluorophore was acquired sequentially at each *z*-step of the confocal to improve the axial precision of the measurements. Nikon NIS-Elements Advanced Research (AR) 5.02 acquisition software was used to acquire the data, and the files were exported in ND2 file format. Figures were generated using Fiji^66^. A Gaussian filter of *σ* = 1 was applied to the image to smooth single pixel noise before adjustment of brightness and contrast. Max intensity projection (MPI) renderings were created by using the 3D projection function (Stacks>3D Project) with 10° increments and interpolation selected to smooth the 3D rendering.

3D image analysis was performed using custom pipelines built in Arivis 4DFusion 4.0 analysis software. We detected VLPs through a particle enhancement denoising filter of diameter 0.6 µm followed by a dilation morphology filter of diameter 0.13 µm (sphere shaped). We then applied a Blob Finder segmentation filter of diameter 0.52 µm, a probability threshold of 21.24%, and a split sensitivity of 50%. We segmented cellular compartments by first applying an enhance edges filter within the membrane detection operation, with a membrane width of 0.9 µm and a gap size of 0.6 µm, to enhance the AF488 signal. A discrete Gaussian denoising filter of diameter 0.2 µm was then applied. The membrane-based segmentation operation with a split sensitivity of 30% and a maximum diameter of 50 µm was executed to segment the processed image, and the whole cell masks were obtained by additional feature filters of sphericity >0.58 and volume >20 µm^3^. The cytoplasm mask was created by eroding the cell mask by two pixels. The membrane + cytoplasm mask was created by dilating the cell mask by three pixels, and the membrane mask was obtained by subtracting the membrane + cytoplasm mask with the cytoplasm mask. Finally, to remove segments created based on cells cut off at the edges of the imaged volume as well as cellular blebs that were segmented as independent cells, we applied a volume filter to exclude cytoplasm segments with a volume of <500 µm^3^ as well as their corresponding membrane segments. The number of VLPs in each compartment was then calculated by combining all masks.

### Biolayer interferometry

Biolayer interferometry was performed using an Octet RED96e (Sartorius) and data were analysed using ForteBio Data Analysis HT version 12.0.1.55 software. PCDH10_EC1_–Fc, VLDLR_LBD_–Fc, and a control IgG (C1A-H12)^65^ were loaded onto Anti-Human IgG Fc Capture (AHC) Biosensors (Sartorius 18-5063) at 100 nM in kinetic buffer (TBS containing 2 mM CaCl_2_ and 0.1% (w/v) BSA) for 80 s. Coated sensor tips were dipped into kinetic buffer for a baseline measurement of 60 s, then dipped into a solution of WEEV VLPs at 100 nM for 300 s (CBA87 VLP) or 600 s (McMillan VLP), and finally in kinetic buffer for dissociation for 300 s (CBA87 VLP) or 600 s (McMillan VLP). In the case of WEEV McMillan VLP, the control IgG (C1A-H12)^65^ was replaced with MXRA8_ect_–Fc as a control.

### Phylogenetic analysis

Sequences encoding the structural polyprotein (C–E3–E2–(6 K/TF)–E1) of 44 WEEV strains with full genome sequences available (Supplementary Table 1) were aligned in MEGA11 using the built-in MUSCLE algorithm^67^. A maximum-likelihood phylogenetic tree was constructed using the aligned sequences with the Tamura 3-parameter nucleotide substitution model. The bootstrap method was used to test phylogeny with 1000 bootstrap replications.

### Replication kinetics assay with authentic WEEV

K562 cells (2.5 × 10^6^) transduced with an empty lentiGuide-Puro vector or transduced to overexpress MXRA8, PCDH10, VLDLR, or ApoER2 were pelleted by centrifugation at 450*g* for 2 min. The medium was discarded and the cell pellets were gently resuspended in 1 ml of solution containing WEEV (strain McMillan or Fleming) diluted to 2.5 × 10^4^ PFU/ml in maintenance medium (RPMI1640 supplemented with 2% (v/v) FBS, 25 mM HEPES, 1% (v/v) penicillin-streptomycin) for a MOI of 0.01. The infection was allowed to proceed for 1 h at 37 °C with 5% CO_2_. Following the infection, the cells were washed three times with 5 ml DPBS (Sigma) by centrifugation as above. Finally, the cells were resuspended in 5 ml maintenance medium. Immediately following this final resuspension, and again at 6, 12, 24 and 48 h post-infection, 500 µl supernatant was collected from each sample and stored at −80 °C. The removed volume was replaced with 500 µl fresh maintenance medium, and the samples were returned to the incubator. Sample titres were determined by plaque assay on Vero E6 cells.

### Plaque assay

Culture supernatants containing WEEV (strain McMillan or Fleming) were serially diluted tenfold in DPBS supplemented with 2% (v/v) FBS. Diluted samples were allowed to infect confluent monolayers of Vero E6 cells in 12-well plates for 1 h at 37 °C with 5% CO_2_. Following the infection, monolayers were overlaid with minimum essential medium (MEM) (Gibco) supplemented with 2% (v/v) FBS, 1% (v/v) GlutaMax (Gibco), 1% sodium bicarbonate (7.5% solution) (Gibco), 1% penicillin-streptomycin, and 0.4% LE agarose (Promega). Infected, overlaid plates were incubated for two days at 37 °C with 5% CO_2_ prior to fixation with 10% buffered formalin (Thermo Fisher). Monolayers were stained with 1% (v/v) crystal violet (Sigma) and plaques were visualized with the aid of a light box.

### Plaque reduction neutralization assay

For plaque neutralization assays with infectious WEEV (strain McMillan), PCDH10_EC1_–Fc or MXRA8_ect_–Fc fusion proteins were serially diluted in PBS supplemented with 2% (v/v) FBS to concentrations of 150–0.073 µg ml^−1^. Diluted proteins (or diluent alone as a control) were combined with an equal volume of virus containing approximately 30 PFU of WEEV McMillan. The mixture of protein and virus was incubated at 37 °C with 5% CO_2_ for one hour. Following this incubation, samples were processed as described for the plaque assay. Per cent neutralization was calculated as follows: per cent neutralization = (1 – (number of plaques in an experimental well/average number of plaques in diluent-only control wells)) × 100%.

### In vivo protection study

Mouse studies were performed as approved by the University of Texas Medical Branch Institutional Animal Care and Use Committee (protocol number 1708051) in accordance with the NIH Guidance for the Care and Use of Laboratory Animals. Mice were fed a 19% protein diet (Teklad, 2919, Irradiated), had 12 h light:dark cycle (06:00–18:00), and were housed in a facility maintained at a temperature range of 20 to 26 °C with a humidity range of 30 to 70%. Food and water were provided ad libitum. Sample sizes for mouse studies were determined based on previously published results for similar in vivo experiments^4^. Randomly assigned mixed-sex cohorts (*n* = 5 female and *n* = 5 male) of 6-week-old CD1 IGS mice (Charles River) received a 50 mg kg^−1^ dose of either PCDH10_EC1_–Fc, control IgG (C1A-H12 anti-SARS-CoV-2 spike antibody)^65^, or PBS via the intraperitoneal route. Six hours later, all mice were infected with 1,000 PFU WEEV McMillan via the subcutaneous route in the left rear footpad. Weights were recorded and health checks were performed daily up to 14 days post-infection. We were not blinded to the treatment or infection status of the mice, also for safety reasons, since WEEV can cause severe disease in humans.

### Statistical analysis

Data were deemed statistically significant when *P* values were <0.05 using version 10 of GraphPad Prism. Experiments were analysed by one-way or two-way ANOVA with multiple comparison correction, or by log-rank (Mantel–Cox) test in GraphPad Prism. *P* values are indicated in each of the figure legends. Statistical methods were not used to predetermine sample sizes.

### Reporting summary

Further information on research design is available in the Nature Portfolio Reporting Summary linked to this article.

## Online content

Any methods, additional references, Nature Portfolio reporting summaries, source data, extended data, supplementary information, acknowledgements, peer review information; details of author contributions and competing interests; and statements of data and code availability are available at 10.1038/s41586-024-07740-2.

### Supplementary information

Supplementary Fig. 1 Uncropped gels for the indicated figures

Reporting Summary

Supplementary Table 1 List of source virus strains tested in this study.

Supplementary Table 2 List of genes and scores from the CRISPR–Cas9 screen after MAGeCK analysis.

### Source data

Source Data Fig. 1

Source Data Fig. 2

Source Data Fig. 3

Source Data Fig. 4

Source Data Fig. 5

Source Data Extended Data Fig. 7

Source Data Extended Data Fig. 8

Source Data Extended Data Fig. 10

Source Data Extended Data Fig. 11

## Data Availability

The list of genes encoding membrane-associated proteins targeted by the CRISPR–Cas9 library are as originally described by Clark et al.^4^. Confocal microscopy images that support the finding of this study are available at https://omero.hms.harvard.edu/webclient/userdata/?experimenter=7554. All other data that support the findings of this study are available within the Article and its Supplementary Information. Source data are provided with this paper.

## Code availability

Custom pipelines built in Arivis 4DFusion 4.0 analysis software used for this study are available at https://github.com/wanyuvirus/Li_et_al.-Arivis.
