# Supplementary material for: Shifts in receptors during submergence of an encephalitic arbovirus
Source: Nature. 2024 Jul 24;632(8025):614–21. doi: 10.1038/s41586-024-07740-2 (PMC11324528; doi:10.1038/s41586-024-07740-2)

nature portfolio

https://doi.org/10.1038/s41586-024-07740-2

Supplementary information

Shifts in receptors during submergence of an encephalitic arbovirus

In the format provided by the authors and unedited

Extended Data Fig. 3a

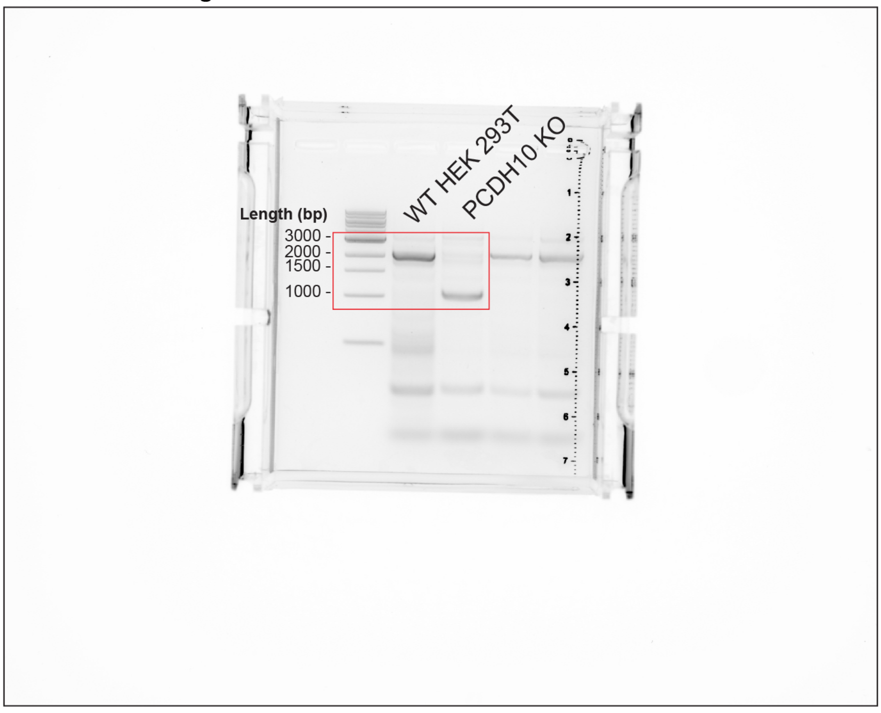

Extended Data Fig. 4d
Extended Data Fig. 4f
Extended Data Fig. 4g

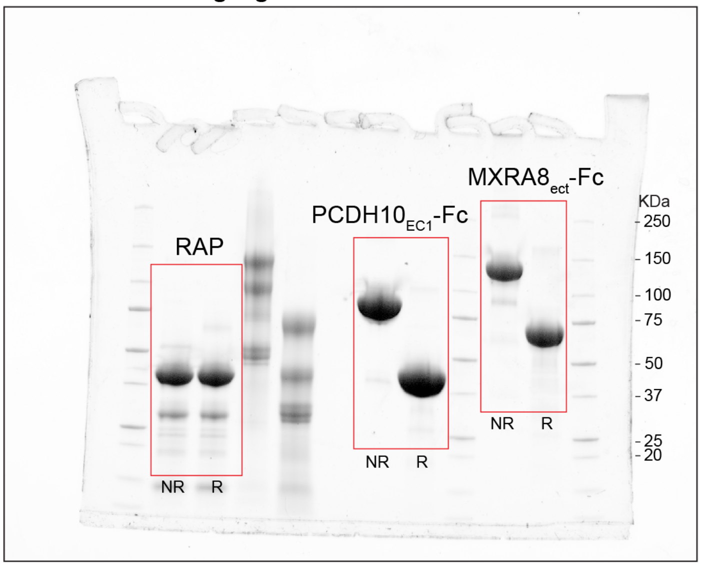

Extended Data Fig. 4e

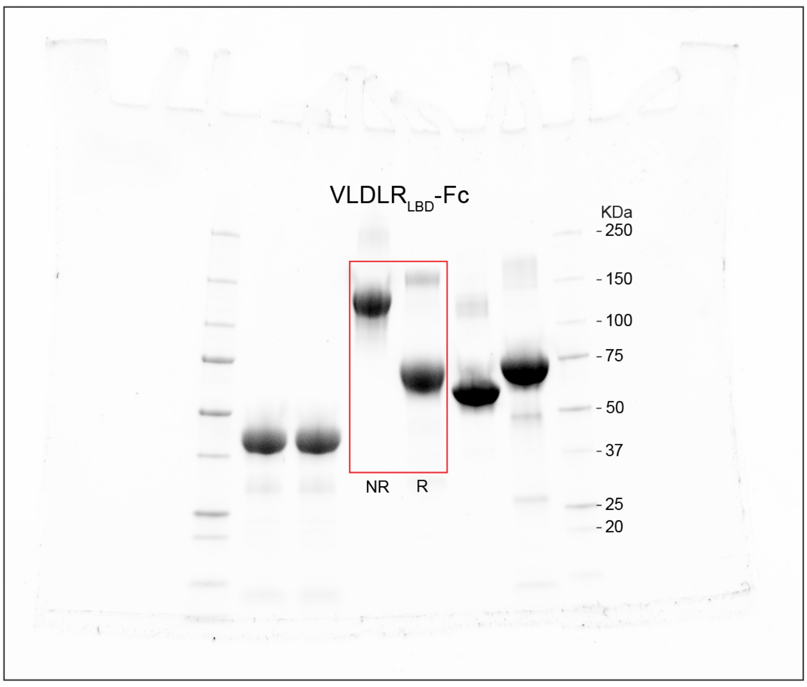

Extended Data Fig. 5a

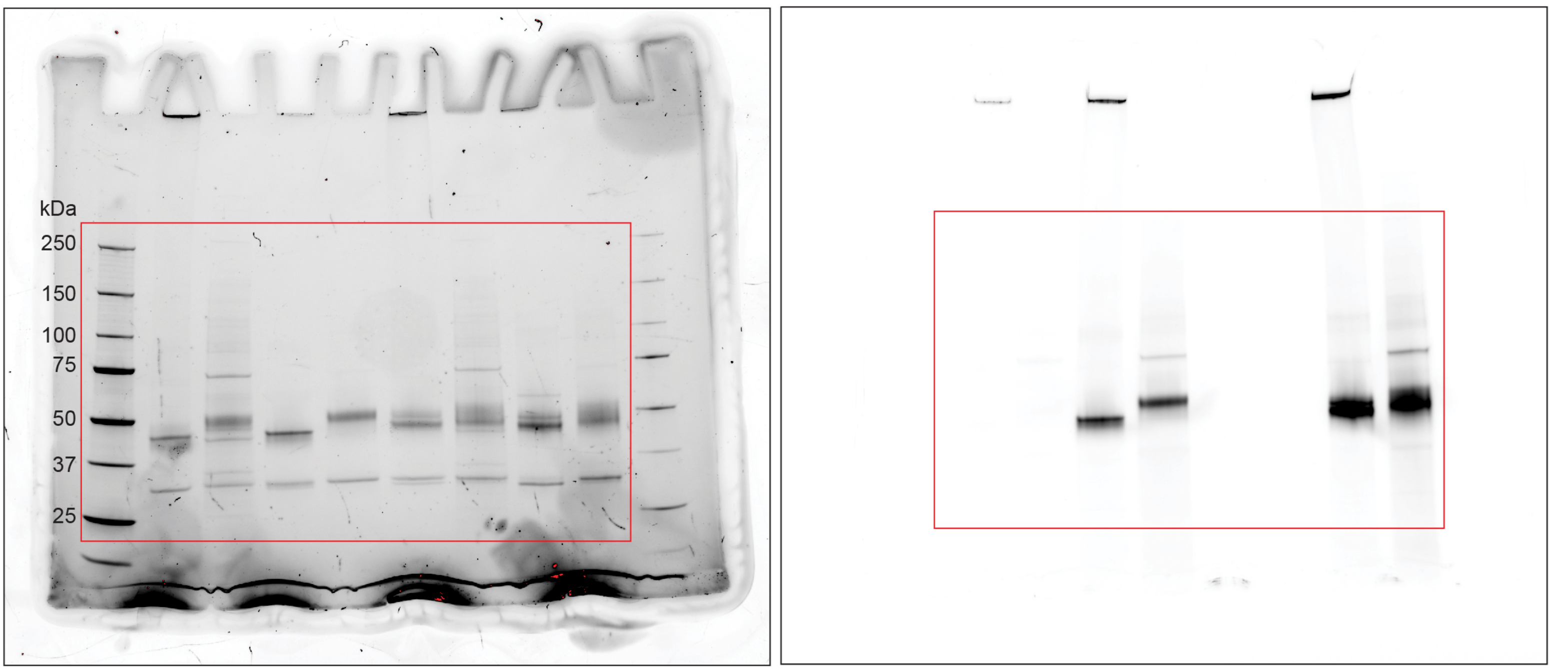

Extended Data Fig. 9a

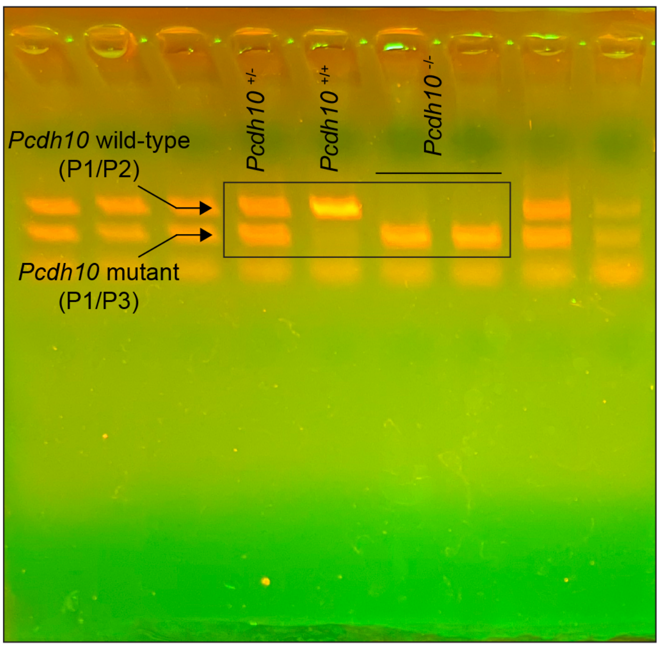

Supplement: Supplementary file 1 — Uncropped gels for the indicated figures [file 41586_2024_7740_MOESM1_ESM.pdf]
