# Supplementary material for: Shifts in receptors during submergence of an encephalitic arbovirus
Source: Nature. 2024 Jul 24;632(8025):614–21. doi: 10.1038/s41586-024-07740-2 (PMC11324528; doi:10.1038/s41586-024-07740-2)
Supplement: Supplementary file 2 — Reporting Summary [file 41586_2024_7740_MOESM2_ESM.pdf]

nature portfolio

Corresponding author(s): Jonathan Abraham

Last updated by author(s): Jun 7, 2024

Reporting Summary

Nature Portfolio wishes to improve the reproducibility of the work that we publish. This form provides structure for consistency and transparency in reporting. For further information on Nature Portfolio policies, see our Editorial Policies and the Editorial Policy Checklist.

Statistics

For all statistical analyses, confirm that the following items are present in the figure legend, table legend, main text, or Methods section.

n/a	Confirmed	
☐	☒	The exact sample size (*n*) for each experimental group/condition, given as a discrete number and unit of measurement
☐	☒	A statement on whether measurements were taken from distinct samples or whether the same sample was measured repeatedly
☐	☒	The statistical test(s) used AND whether they are one- or two-sided<br>*Only common tests should be described solely by name; describe more complex techniques in the Methods section.*
☐	☒	A description of all covariates tested
☐	☒	A description of any assumptions or corrections, such as tests of normality and adjustment for multiple comparisons
☐	☒	A full description of the statistical parameters including central tendency (e.g. means) or other basic estimates (e.g. regression coefficient) AND variation (e.g. standard deviation) or associated estimates of uncertainty (e.g. confidence intervals)
☐	☒	For null hypothesis testing, the test statistic (e.g. *F*, *t*, *r*) with confidence intervals, effect sizes, degrees of freedom and *P* value noted<br>*Give P values as exact values whenever suitable.*
☒	☐	For Bayesian analysis, information on the choice of priors and Markov chain Monte Carlo settings
☒	☐	For hierarchical and complex designs, identification of the appropriate level for tests and full reporting of outcomes
☒	☐	Estimates of effect sizes (e.g. Cohen's *d*, Pearson's *r*), indicating how they were calculated

Our web collection on statistics for biologists contains articles on many of the points above.

Software and code

Policy information about availability of computer code

Data collection	IntelliCyt ForeCyt Standard Edition Version 8.1.7524, ForteBio Data Analysis HT Version 12.0.1.55, Nikon NIS-Elements Advanced Research (AR) 5.02, Incucyte S3 Software (v2022B Rev2)
Data analysis	MAGeCK version 0.5.6, FlowJo version 10.6.2, GraphPad Prism (version 10.1.0), Arivis Vision4D version 4.0, Incucyte S3 Software (v2022B Rev2), MEGA (version 11.0.10), MUSCLE algorithm (web-based at EMBL-EBI), MUSCLE algorithm (MEGA 11 built-in)

For manuscripts utilizing custom algorithms or software that are central to the research but not yet described in published literature, software must be made available to editors and reviewers. We strongly encourage code deposition in a community repository (e.g. GitHub). See the Nature Portfolio guidelines for submitting code & software for further information.

Data

Policy information about availability of data

All manuscripts must include a data availability statement. This statement should provide the following information, where applicable:

- Accession codes, unique identifiers, or web links for publicly available datasets
- A description of any restrictions on data availability
- For clinical datasets or third party data, please ensure that the statement adheres to our policy

The list of genes encoding membrane-associated proteins targeted by the CRISPR–Cas9 library are as originally described by Clark et al.(PMID: 34929721). Confocal

microscopy images that support the finding of this study are available at https://omero.hms.harvard.edu/webclient/userdata/?experimenter=7554. All other data that support the findings of this study are available within the Article and its Supplementary Information. Source data are provided.

Research involving human participants, their data, or biological material

Policy information about studies with human participants or human data. See also policy information about sex, gender (identity/presentation), and sexual orientation and race, ethnicity and racism.

Reporting on sex and gender	N/A
Reporting on race, ethnicity, or other socially relevant groupings	N/A
Population characteristics	N/A
Recruitment	N/A
Ethics oversight	N/A

Note that full information on the approval of the study protocol must also be provided in the manuscript.

Field-specific reporting

Please select the one below that is the best fit for your research. If you are not sure, read the appropriate sections before making your selection.

☒ Life sciences ☐ Behavioural & social sciences ☐ Ecological, evolutionary & environmental sciences

For a reference copy of the document with all sections, see nature.com/documents/nr-reporting-summary-flat.pdf

Life sciences study design

All studies must disclose on these points even when the disclosure is negative.

Sample size	Sample sizes for mouse studies were determined based on previously published results for similar in vivo experiments (PMID: 34929721). No sample size calculations were performed to power each study and no statistical methods were used to predetermine sample size.
Data exclusions	For confocal microscopy experiments, after segmentation of cytoplasm and membrane compartments and quantification of VLPs in each compartment, a volume filter was applied to remove cytoplasm compartments that are less than 500 µm^3 in size and their corresponding membrane compartments, and VLPs in those removed compartments were not counted. The volume filter was applied because upon examination of segmented micrographs, we found that these small cytoplasm segments corresponded to cellular blebs that were segmented as independent cells, or to cells cut off at the edge of the imaged volume. Segments of the latter case were excluded because internalized VLPs appear to be locally concentrated within the cell and not even distributed throughout the cytoplasm, so partial cells at the edge of the imaged volume may not accurately reflect presence of VLPs. The filter threshold is set well below the average size of K562 cells (PMID: 6996765) so is expected to preserve intact cells. This exclusion criterion was not pre-established. No other data were excluded.
Replication	Cell-based experiments were performed at least twice independently, each containing at least duplicates for every treatment. n values are defined and provided in each figure legend. All attempts to replicate results were successful.
Randomization	For cell-based studies and biolayer interferometry experiments, sample allocation was not randomized because the results are quantitative and did not require subjective judgment or interpretation. This practice is standard in the field (PMID: 33208938). For mouse experiments, mice were randomly assigned to mixed-sex cohorts (male n=5, female n=5). Male and female mice had different average body weights and therefore received different amounts of recombinant proteins according to the sex-specific average weight to achieve comparable dosage.
Blinding	The investigators were not blinded to the allocation during experiments or to outcome assessment for in vivo or in vitro studies. Blinding was not deemed necessary because the results are quantitative and did not require subjective judgment or interpretation. Blinding is also not typically used in the field for similar in vitro and in vivo studies (PMID: 33208938). For in vivo studies, researchers were not blinded to the treatment or infection status of the mice also for safety reasons, because WEEV can cause severe disease in humans.

Reporting for specific materials, systems and methods

We require information from authors about some types of materials, experimental systems and methods used in many studies. Here, indicate whether each material, system or method listed is relevant to your study. If you are not sure if a list item applies to your research, read the appropriate section before selecting a response.

April 2023

Materials & experimental systems

n/a	Involved in the study
☐	☒ Antibodies
☐	☒ Eukaryotic cell lines
☒	☐ Palaeontology and archaeology
☐	☒ Animals and other organisms
☒	☐ Clinical data
☒	☐ Dual use research of concern
☒	☐ Plants

Methods

n/a	Involved in the study
☒	☐ ChIP-seq
☐	☒ Flow cytometry
☒	☐ MRI-based neuroimaging

Antibodies

Antibodies used	anti-PCDH10 antibody (Proteintech 21859-1-AP), anti-HLA-C antibody (Proteintech 15777-1-AP), anti-VLDLR antibody (clone 1H10, GeneTex GTX79552), anti-MXRA8 antibody (clone 2H2G12A, MBL International W040-3), anti-LDLR antibody (clone 472413, R&D Systems MAB2148), rabbit IgG isotype (Proteintech 30000-0-AP), mouse IgG isotype (clone MPC-11, BD Biosciences BDB557351), PE-conjugated donkey anti-rabbit F(ab')2 fragment (Jackson ImmunoResearch 711-116-152), PE-conjugated donkey anti-mouse F(ab')2 fragment (Jackson ImmunoResearch 715-116-150), APC-conjugated rat anti-DYKDDDDK (Flag) antibody (clone L5, BioLegend 637307), APC-conjugated rat isotype control antibody (BioLegend 402306), anti-CD20 APC conjugate antibody (Clone LT20, Miltenyi Biotec #130-113-370), anti-HLA-ABC antibody (Proteintech 15240-1-AP).
Validation	1. anti-PCDH10 antibody (Proteintech 21859-1-AP): commercially validated, tested applications are western blot, immunohistochemistry, immunofluorescence, with human and mouse reactivity.<br>2. anti-VLDLR antibody (GeneTex GTX79552): commercially validated, tested applications are western blot, immunocytochemistry/ immunofluorescence, flow cytometry, ELISA, with human reactivity.<br>3. anti-MXRA8 antibody (MBL International W040-3): commercially validated, tested applications include flow cytometry, with human reactivity<br>4. rabbit IgG isotype (Proteintech 30000-0-AP): commercially validated, tested applications include western blot, immunoprecipitation, flow cytometry, cited reactivity includes human, mouse, rat, sheep.<br>5. mouse IgG isotype (BD Biosciences BDB557351): commercially validated, tested applications include immunohistochemistry, flow cytometry. Routinely tested with ELISA.<br>6. PE-conjugated donkey anti-rabbit F(ab')2 fragment (Jackson ImmunoResearch 711-116-152): ommercially validated, reacts with whole molecule rabbit IgG and the light chains of other rabbit immunoglobulins, tested applications include ELISA, minimal cross-reaction with bovine, chicken, goat, guinea pig, syrian hamster, horse, human, mouse, rat and sheep serum proteins, but it may cross-react with immunoglobulins from other species.<br>7. PE-conjugated donkey anti-mouse F(ab')2 fragment (Jackson ImmunoResearch 715-116-150): commercially validated, reacts with whole molecule mouse IgG and the light chains of other mouse immunoglobulins, tested applications include ELISA, minimal cross-reaction with bovine, chicken, goat, guinea pig, syrian hamster, horse, human, rabbit and sheep serum proteins, but may cross-react with immunoglobulins from other species.<br>8. APC-conjugated rat anti-DYKDDDDK (Flag) antibody (BioLegend 637307): commercially validated via intracellular immunofluorescent staining and flow cytometry.<br>9. APC-conjugated rat IgG2a, λ Isotype Ctrl Antibody (BioLegend 402306): commercially validated via cell surface immunofluorescent staining and flow cytometry.<br>10. anti-CD20 APC conjugate antibody (Miltenyi Biotec, Clone LT20 #130-113-370): commercially validated, extended validation performed through epitope competition assays with other known clones recognizing the same antigen, application staining of formaldehyde-fixed cells, immunofluorescence, immunohistochemistry, immunocytochemistry, reactivity human.<br>11. anti-LDLR antibody (R&D Systems MAB2148): commercially validated, tested applications include western blot, immunofluorescence, flow cytometry, and ELISA, with no cross-reactivity with mouse LDLR.<br>12. anti-HLA-C antibody (Proteintech 15777-1-AP): commercially validated, tested applications include western blot, immunoprecipitation, immunohistochemistry, immunofluorescence, with human reactivity.<br>13. anti-HLA-ABC antibody (Proteintech 15240-1-AP): commerically validated, tested applications include western blot, immunoprecipitation, immunohistochemistry, immunofluorescence, with human reactivity.

Eukaryotic cell lines

Policy information about cell lines and Sex and Gender in Research

Cell line source(s)	HEK 293T (human kidney epithelial, ATCC CRL-11268), Vero E6 (Cercopithecus aethiops, ATCC CRL-1586), Vero 81 (ATCC CCL-81), SVG-A (human astroglial, provided by T. Kirchhausen and invented by W.J. Atwood, not from commerical sources), K562 (human chronic myelogenous leukemia, ATCC CCL-243), SK-N-SH (human brain, ATCC HTB-11), Expi293F cells (Thermo Fisher Scientific A14527).
Authentication	Cell lines were not authenticated. All cell lines grew as expected and had the expected morphology when inspected by microscopy.
Mycoplasma contamination	We confirmed the absence of mycoplasma in all cell lines through monthly testing using an e-Myco PCR detection kit (Bulldog Bio).
Commonly misidentified lines (See ICLAC register)	None.

Animals and other research organisms

Policy information about studies involving animals; ARRIVE guidelines recommended for reporting animal research, and Sex and Gender in Research

Laboratory animals	For in vivo protection studies, six-week old CD-1 mice were used. Each cohort contains 5 male mice and 5 female mice. Mice were fed a 19% protein diet (Teklad, 2919, Irradiated), had 12 h light/dark cycle (0600-1800), and were housed in a facility maintained at a temperature range of 20-26 °C with a humidity range of 30–70. Food and water were provided ad libitum. For mouse cortical neuron isolation, postnatal day 1 or day 2 C57BL/6J mice were used. Cortices derived from individual pups were processed separately, and we did not keep track of exact sexes of each pup, which is challenging to do in neonatal ages (therefore, analysis likely included both male and female mice).
Wild animals	The study did not involve wild animals.
Reporting on sex	Each cohort contains 5 male mice and 5 female mice. The amounts of injected recombinant proteins were equalized based on the average weight of male or female mice, so that individuals receive comparable doses. Data were not disaggregated for sex.
Field-collected samples	No field-involved samples were collected in this study.
Ethics oversight	Mouse experiments were approved at Harvard Medical School under the Harvard Medical School Institutional Animal Care and Use Committee (protocol number IS00002530-3), the Boston Children's Hospital Institutional Animal Care and Use Committee (protocol number 00001725), and the University of Texas Medical Branch School Institutional Animal Care and Use Committee (protocol number 1708051)

Note that full information on the approval of the study protocol must also be provided in the manuscript.

Plants

Seed stocks	N/A
Novel plant genotypes	N/A
Authentication	N/A

Flow Cytometry

Plots

Confirm that:

- [x] The axis labels state the marker and fluorochrome used (e.g. CD4-FITC).
- [x] The axis scales are clearly visible. Include numbers along axes only for bottom left plot of group (a 'group' is an analysis of identical markers).
- [x] All plots are contour plots with outliers or pseudocolor plots.
- [x] A numerical value for number of cells or percentage (with statistics) is provided.

Methodology

Sample preparation	Primary antibodies were diluted to 10 µg ml−1 in binding buffer (2% (v/v) goat serum in PBS) immediately before use. Cells were incubated in blocking buffer (5% [v/v] goat serum in PBS) for 30 min at 4°C followed by incubation with primary antibodies (rabbit anti-PCDH10 antibody (Proteintech 21859-1-AP), mouse anti-VLDLR antibody (GeneTex GTX79552), mouse anti-MXRA8 antibody (MBL International W040-3), mouse anti-LDLR antibody (R&D Systems MAB2148)) in binding buffer (2% [v/v] goat serum in PBS). Cells were washed three times in binding buffer and subsequently incubated with a PE-conjugated donkey anti-rabbit F(ab')2 fragment (Jackson ImmunoResearch 711-116-152) or a PE-conjugated donkey anti-mouse F(ab')2 fragment (Jackson ImmunoResearch 715-116-150) according to the host species of the primary antibodies, diluted 1:200 in binding buffer for 30 min at 4°C. We washed cells twice in binding buffer and twice in PBS, fixed cells in 2% [v/v] formalin, and detected cell surface receptor expression using flow cytometry.<br>For cells expressing ApoER2 isoform 2, we used recombinant Flag-tagged RAP as a substitute for primary antibody. The secondary antibody used was an APC-conjugated anti-DYKDDDDK (Flag) antibody (BioLegend 637307).<br>For immunostaining of cells expressing Flag-tagged receptors, we added an APC-conjugated rat anti-DYKDDDDK (anti-Flag) antibody (BioLegend Cat#: 637307) or an isotype control antibody (BioLegend Cat#: 402306) in binding buffer. Cells were incubated in blocking buffer for 30 min at 4 °C. After one more wash with binding buffer, cells were incubated with the antibodies for 30 min at 4 °C. Following incubation, we washed cells twice with binding buffer, twice with PBS, fixed them

	with 2% [v/v] formalin, and detected cell surface receptor expression by flow cytometry.<br>For experiments with GFP-expressing alphavirus reporter virus particles, twenty four hours post-infection, cells were harvested, washed twice with phosphate buffered saline (PBS), and fixed in PBS containing 2% (v/v) formalin. GFP expression was measured by flow cytometry.
Instrument	iQue3 Screener PLUS (Intellicyt).
Software	IntelliCyt ForeCyt Standard Edition version 8.1.7524 (Sartorius).
Cell population abundance	For sorted stable cell lines, purity was confirmed by cell surface staining after expansion in media containing puromycin.
Gating strategy	Gated for live cells with FSC-H and SSC-H. Gated for single cells with FSC-H and FSC-A. Then gated for GFP positive or fluorophore (PE or APC) positive cells.

☒ Tick this box to confirm that a figure exemplifying the gating strategy is provided in the Supplementary Information.
